# Hierarchy of Relaxation Times and Residual Entropy: A Nonequilibrium Approach

**DOI:** 10.3390/e20030149

**Published:** 2018-02-26

**Authors:** Purushottam D. Gujrati

**Affiliations:** 1 Department of Physics, The University of Akron, Akron, OH 44325, USA; pdg@uakron.edu; 2 Department of Polymer Science, The University of Akron, Akron, OH 44325, USA

**Keywords:** relaxation hierarchy, residual entropy, nonequilibrium thermodynamics, internal variables, hierarchy of state spaces, tool-narayanaswamy equation, entropy bound

## Abstract

We consider nonequilibrium (NEQ) states such as supercooled liquids and glasses that are described with the use of internal variables. We classify the latter by the state-dependent hierarchy of relaxation times to assess their relevance for irreversible contributions. Given an observation time $\tau_{\mathrm{obs}}$, we determine the window of relaxation times that divide the internal variables into active and inactive groups, the former playing a central role in the NEQ thermodynamics. Using this thermodynamics, we determine (i) a bound on the NEQ entropy and on the residual entropy and (ii) the nature of the isothermal relaxation of the entropy and the enthalpy in accordance with the second law. A theory that violates the second law such as the entropy loss view is shown to be internally inconsistent if we require it to be consistent with experiments. The inactive internal variables still play an indirect role in determining the temperature T(t) and the pressure P(t) of the system, which deviate from their external values.

## 1. Introduction

**Figure 1:**
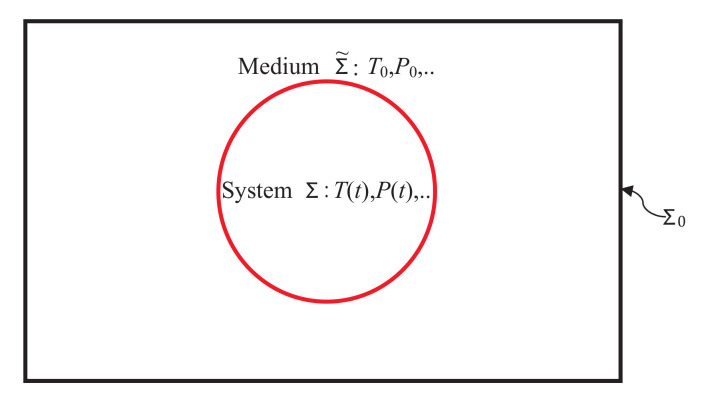
An isolated system $\Sigma_{0}$ consisting of the system Σ in a surrounding medium $\tilde{\Sigma }$. The medium and the system are characterized by their fields $T_{0},P_{0},\ldots$ and T(t),P(t),…, respectively, which are different when the two are out of equilibrium.

Glass, such as naturally-occurring obsidian, pumice, etc., or man-made Venetian glass, window glass, etc., is a well-known class of materials that has captured our fascination forever. We can now make a defect-free glass in the laboratory for a variety of scientific and technological applications. Crudely speaking, it is an almost solid-like amorphous material that possesses no long-range atomic order and, upon heating, gradually softens as it turns into its molten state (also known as the supercooled liquid) as it passes through the glass transition region normally denoted by a suitable chosen single temperature $T_{g}$ in this region [1,2,3,4]. For the purpose of this article, a glass is treated merely as a nonequilibrium (NEQ) state of matter, which can be made quite homogeneous so to a good approximation, it can be treated as a thermodynamic system that is in internal equilibrium (IEQ), but not in equilibrium (EQ), as explained later (at present, it suffices to say that the entropy in an IEQ state is a state function of its state variables that now include some NEQ state variables (commonly known as internal variables) [1,2,3,4] besides those needed to specify EQ states; see also [5,6,7]). This means that a glass will exhibit relaxation as it strives to come to equilibrium. The relaxation time is known to be large enough close to $T_{g}$ that at much lower temperatures, one can usually treat a glass as in an almost frozen state over experimental time scale $\tau_{\mathrm{obs}}$, the time period over which successive observations are made. We refer the reader to an excellent monograph by Debenedetti [3] on these issues. We will primarily focus on the thermodynamics of glasses and supercooled liquid in this work and treat them as NEQ states. Therefore, our discussion will mostly consider a NEQ system, which we denote by Σ in an extensively large medium $\tilde{\Sigma }$ as shown in Figure 1.

**Definition** **1.** *As we will not consider a system in isolation in this work, we will always use EQ or “equilibrium” to mean “equilibrium with respect to the medium $\tilde{\Sigma }$.” We will not reserve EQ for the entire system only. We will also use it for a part of the system, part of the state variables or part of the degrees of freedom such as vibrational degrees of the system, if they are in equilibrium with $\tilde{\Sigma }$. On the other hand, we will reserve the use of IEQ for the entire system; see also [5].*

It is a well-known fact that in glasses, the vibrational modes come to equilibrium very fast, even though the glass is out of equilibrium. Similarly, in a sinusoidal variation of *T*, some degrees of freedom would equilibrate after a cycle; others would not and would control the temporal behavior of the system. It seems natural that the sinusoidal variation would give rise to a distribution of relaxation times. Thus, in general, one of the most important consequences of the rate of variation of the external stimuli such as the temperature or pressure is the possibility that the state of the system may be so far away from equilibrium that the dynamics becomes too complex, involving multiple relaxation time scales $\tau_{0},\tau_{1},\tau_{2},\ldots$, in supercooled liquids [1,3,8,9]. The relaxation time is defined as the time required for the corresponding dynamical variable to come to equilibrium with the medium; see Equation (7) for the proper definition of the relaxation time. It should be emphasized that this interpretation of the relaxation time is dictated by the experimental setup, but does not depend on any particular mathematical form of the relaxation. An interplay between $\tau_{\mathrm{obs}}$ and relaxation times $\tau_{k}$’s becomes crucial in determining the thermodynamics of the system and plays a major role in our discussion here. In fact, one of the following cases for a given $\tau_{k}$ will be usually encountered in experiments:

Relax 1 $\tau_{k}<<\tau_{\mathrm{obs}}$: In this situation, the *k*-th relaxing dynamical variable has equilibrated and does not have to be accounted for in the NEQ thermodynamics.
Relax 2 $\tau_{k}≃\tau_{\mathrm{obs}}$: In this situation, the *k*-th dynamical variable will continue to relax towards equilibrium during $\tau_{\mathrm{obs}}$ and must be accounted for as the system approaches equilibrium.
Relax 3 $\tau_{k}>>\tau_{\mathrm{obs}}$: In this situation, the *k*-th dynamical variable will not fully relax and will strongly affect the behavior of the system. The corresponding dynamical variable is said to be “frozen-in” over $\tau_{\mathrm{obs}}$.

When there are several relaxation times, it is possible that different $\tau_{k}$’s will correspond to different cases above. Thus, care must be exercised in dealing with different relaxation times. The need for such care has been recognized in vitrification for a long time [10]. Relaxation is a universal phenomenon when a system drives itself towards a more stable state such as an EQ state. In liquids or glasses, relaxations involving changes of the atomic or molecular positions are generally known as structural relaxations [11]. Recent experimentation advances have made it possible to directly measure these relaxation processes at the molecular level simultaneously [12]. At sufficiently low temperatures, the characteristic time for structural relaxations becomes comparable to the time scale of a macroscopic observation $\tau_{\mathrm{obs}}∼$ 100 s. For shorter time scales, the supercooled liquid (SCL) exhibits solid-like properties, while for longer times, it shows liquid-like properties. Even the dynamics in these cases is not so trivial, but has been investigated for a long time [1,3,8,9] with tremendous success. The glass transition being an “NEQ transition,” its description will require extensive internal variables, collectively denoted by a vector ξ that are independent of the set X of extensive observables (E,V,N,…) [13] whenever the system is out of equilibrium [2,4,14,15,16,17,18,19,20,21,22,23,24,25]. We denote their collection by Z in this work. The investigations of the glass transition invariably assume that the entropy *S* is a state function S(Z) of the state variables in the extended state space $S_{Z}⊃S_{X}$ spanned by Z; here, $S_{X}$ is the state space of the observables. There is a memory of the initial state, and it requires the entire history of how the state is prepared to uniquely describe the preparation. Such a memory in some cases can be described by ξ. One example is residual stresses [26]: if particle configurations in a glass cannot fully relax to equilibrium, some of the stresses that build up during flow in the melt persist in the glass; these stresses cannot be captured by X. We will say that such a state is an incompletely described state in terms of X. but a completely described state in terms of Z. In contrast, the EQ state $M_{\mathrm{eq}}\left(X\right)$ is a completely (i.e., uniquely) described state by X and has no memory of the initial state. This means that in equilibrium, ξ is no longer independent of X.

The consideration of dynamics resulting from the simple connectivity of the sample (also known as the microstate or phase) space has played a pivotal role in developing the kinetic theory of gases [27,28,29], where the interest is at high temperatures [7,30,31,32]. As the dynamics is very fast here, it is well known that the ensemble averages agree with temporal averages. However, at low temperatures, where dynamics becomes sluggish as in a glass [3,33,34,35], the system can be confined into disjoint components. The confinement occurs under NEQ conditions, when the observational time scale $\tau_{\mathrm{obs}}$ becomes shorter than the equilibration time $\tau_{\mathrm{eq}}$, such as in glasses, whose behavior and properties have been extensively studied. These components are commonly known as basins in the energy landscape picture [36,37]. The entropy of confinement at absolute zero is known as the residual entropy and can be observed in glasses or disordered crystals; see below.

The existence of a nonzero residual entropy does not violate Nernst’s postulate, as the latter is applicable only to EQ states [7] (Section 64). The observation of residual entropy is very common in Nature. Indeed, Tolman [38] (Section 137) devotes an entire section on this issue for crystals in his seminal work, while Sethna provides an illuminating discussion for glasses [39] (Section 5.2.2). In addition, the existence of the residual entropy has been demonstrated rigorously for glasses by Pauli and Tolman [40] and for a very general spin model by Chow and Wu [41]; see the references in these works for other cases where the residual entropy is shown to exist rigorously. The numerical simulation carried out by Bowles and Speedy for glassy dimers [42] also supports the existence of a residual entropy. We refer the reader to consult various publications [38,43,44]. Experiment evidence for a nonzero residual entropy is abundant as discussed by several authors [30,35,42,45,46,47,48,49,50,51]; various textbooks [2,4] also discuss this issue.

We introduce useful notation and concepts in the next section. In the following section, we introduce the concept of internal equilibrium (IEQ) states for which the entropy is a state function in the extended state space $S_{Z}$.

**Definition** **2.** *As we are not interested in ordering phenomena (such as crystallization), we define an NEQ state with respect to an EQ state that is also disordered, i.e., with respect to SCL. This is formally done by considering only disordered configurations and discarding all ordered configurations in our discussion. We warn the reader that this is different from the conventional approach in which the equilibrium state is always taken to be the perfectly crystalline state. This point should not be forgotten. We then discuss the nature of the nonequilibrium state variables in $S_{Z}$ in Proposition 1. The affinity A corresponding to **ξ** is defined so that it vanishes in SCL, the equilibrium state in our approach.*

The concept of a hierarchy of relaxation times is introduced in Section 4, which forms a central part of the paper. A given $\tau_{\mathrm{obs}}$ determines a particular time window, which provides a justification for Proposition 1. We find that internal variable $\xi_{E}$ that has equilibrated plays no role thermodynamically since the affinity vanishes during $\tau_{\mathrm{obs}}$. In Section 5, we discuss the first law in terms of the new notation, identify the irreversible work and the IEQ thermodynamics to be used in the next two sections on the entropy bound in vitrification and the residual entropy (Section 6) and on the properties of the isothermal relaxation (7). In Section 8, we find that $\xi_{E}$ still indirectly affects the thermodynamics as it is required to have a thermodynamic temperature, pressure, etc., for the system. The final section contains a brief discussion of the results.

## 2. Notation

Below is a brief introduction to the notation and the significance of various modern terminology [19,21] for readers who are unfamiliar with them. As usual, Σ and $\tilde{\Sigma }$ form an isolated system $\Sigma_{0}$. Extensive quantities associated with $\tilde{\Sigma }$ and $\Sigma_{0}$ carry a tilde $\tilde{□}$ and a suffix 0, respectively. As $\tilde{\Sigma }$ is very large compared to Σ and is in equilibrium, all its conjugate fields $T_{0},P_{0},$ etc., carry a suffix 0 as they are the same as for $\Sigma_{0}$, and there is no irreversibility in $\tilde{\Sigma }$. Any irreversibility is ascribed to the system Σ [19,21] and is caused by processes such as dissipation due to viscosity, internal inhomogeneities, etc., that are internal to the system. Quantities without any suffix refer to the system. Throughout this work, we will assume that Σ and $\tilde{\Sigma }$ are spatially disjoint and statistically quasi-independent [25,52,53] so that their volumes, masses and entropies are additive at each instant. In particular, $d\tilde{V}=-dV$, since $V_{0}=V+\tilde{V}$ remains constant for $\Sigma_{0}$. We define a quantity to be a system-intrinsic (SI) quantity if it depends only on the property of the system alone and nothing else. For example, if *P* is the pressure of Σ and $P_{0}$ that of $\tilde{\Sigma }$, then PdV is the SI work done by the system, but $P_{0}dV$ is not as the latter also depends on $\tilde{\Sigma }$ through $P_{0}$. However, $P_{0}d\tilde{V}=-P_{0}dV$ is the work done by the medium, and this work can be identified as a medium-intrinsic (MI) quantity. Any extensive SI quantity q(t) of Σ can undergo two distinct kinds of changes in time: one due to the exchange with the medium and another one due to internal processes. Following modern notation [19,21], exchanges of q(t) with the medium and changes within the system carry the suffixes e and i, respectively:

(1) $$dq\left(t\right)≐q\left(t+dt\right)-q\left(t\right)\equiv d_{e}q\left(t\right)+d_{i}q\left(t\right).$$

For $\tilde{\Sigma }$ and $\Sigma_{0}$, we must replace q(t) by $\tilde{q}\left(t\right)$ and $q_{0}\left(t\right)$, respectively, so that $d\tilde{q}\left(t\right)=\tilde{q}\left(t+dt\right)-\tilde{q}\left(t\right)$ and $dq_{0}\left(t\right)≐q_{0}\left(t+dt\right)-q_{0}\left(t\right)$. We will assume additivity so that:

$$q_{0}\left(t\right)=q\left(t\right)+\tilde{q}\left(t\right).$$

For this to hold, we need to assume that Σ and $\tilde{\Sigma }$ interact so weakly that their interactions can be neglected. As there is no irreversibility within $\tilde{\Sigma }$, we must have $d_{i}\tilde{q}\left(t\right)=0$ for any medium quantity $\tilde{q}\left(t\right)$ and:

(2) $$d_{e}q\left(t\right)≐-d\tilde{q}\left(t\right)=-d_{e}\tilde{q}\left(t\right).$$

It follows from additivity that:

(3) $$dq_{0}\left(t\right)\equiv dq\left(t\right)+d\tilde{q}\left(t\right)=d_{i}q\left(t\right).$$

This means that any irreversibility in $\Sigma_{0}$ is ascribed to Σ, and not to $\tilde{\Sigma }$. In a reversible change, $d_{i}q\left(t\right)\equiv 0$. For example, the entropy change:

$$dS\equiv d_{e}S+d_{i}S$$

for Σ; here,

$$d_{e}S=-d_{e}\tilde{S}$$

is the entropy exchange with the medium and $d_{i}S$ is the irreversible entropy generation due to internal processes within Σ; the latter is also the entropy change $dS_{0}$ of $\Sigma_{0}$; see Equation (3). Similarly, if dW and dQ represent the work done by and the heat change of the system, then:

(4) $$dW\equiv d_{e}W+d_{i}W,dQ\equiv d_{e}Q+d_{i}Q.$$

Here, $d_{e}W$ and $d_{e}Q$ are the work exchange and heat exchange with the medium, respectively, and $d_{i}W\;$ and $d_{i}Q$ are irreversible work done and heat generation due to internal processes in Σ. For an isolated system such as $\Sigma_{0}$, the exchange quantity vanishes so that:

(5) $$dW_{0}\left(t\right)=d_{i}W_{0}\left(t\right);dQ_{0}\left(t\right)=d_{i}Q_{0}\left(t\right).$$

We have introduced the pressure-volume work. We identify $d_{e}W=P_{0}dV=-d_{e}\tilde{W},dW=PdV$ and $d_{i}W=\left(P-P_{0}\right)dV$. In the absence of any chemical reaction, $dN_{k}=d_{e}N_{k},d_{i}N_{k}=0$ for the *k*-th species of the particles; otherwise, $d_{i}N_{k}$ is its change due to the chemical reaction within Σ. As the energy of Σ can only change due to exchange with $\tilde{\Sigma }$,

(6) $$dE=d_{e}E,d_{i}E=0.$$

We now explain the concept of the relaxation time used in this work, which is a simple generalization of its common usage, but which proves useful here. Consider some dynamical variable Φ(t) as a function of time. Its dependence on Z(t) is suppressed. Let Φ(∞) denote its limiting value as t→∞; thus, it also represents its EQ value. In reality, we do not have to wait an infinite amount of time as we cannot distinguish between a nonzero difference $\left|\Phi (t)-\Phi (\infty )\right|$, which is smaller than some small cutoff value so that for all purposes it is no different than zero, or a zero difference. Let us introduce a normalized ratio:

$$\varphi \left(t\right)=\left|[\Phi (t)-\Phi (\infty )]/[\Phi (0)-\Phi (\infty )]\right|$$

to account for this cutoff value, which we denote by $e^{-\lambda }>0$; the cutoff is primarily determined by the experimental setup. We say that the dynamical variable Φ(t) has equilibrated when φ(t) equals the cutoff $e^{-\lambda }$. The relaxation time $\tau_{\mathrm{rel}}$ is defined by:

(7) $$\varphi \left(\tau_{\mathrm{rel}}\right)=e^{-\lambda }.$$

It is clear that for a given choice λ, the relaxation time $\tau_{\mathrm{rel}}$ can be used to describe how rapidly a quantity effectively reaches its equilibrium value. Usually, one assumes for φ(t) an exponential form:

φ(t)=exp(−t/τ)

or a stretched exponential form:

$$\varphi \left(t\right)=\exp\left(-{\left(t/\tau \right)}^{\beta }\right),0<\beta \leq 1,$$

also known as the Kohlrausch–Williams–Watts form, which reduces to the simple exponential for β=1. The relaxation time is:

(8) $$\tau_{\mathrm{rel}}=\lambda^{1/\beta }\tau ,$$

and reduces to $\tau_{\mathrm{rel}}=\lambda \tau$ for β=1, the exponential form. In this work, we do not make any particular choice for the decay behavior of φ(t); thus, we do not make any distinction between the two forms of relaxation given above or any other form. We use a similar cutoff to identify the equilibration time $\tau_{\mathrm{eq}}$. In reality, the stretched exponential is very common in glassy dynamics, but its origin is far from clear at present, even though attempts have been made to express it as a superposition of simple exponentials with different τ’s [54,55]. It is, therefore, treated as empirical in nature. The origin for the exponential relaxation, on the other hand, is well known as the Debye dynamics. For us, what is important is the existence of $\tau_{\mathrm{rel}}$ through Equation (7) and not the actual form of φ(t).

**Figure 2:**
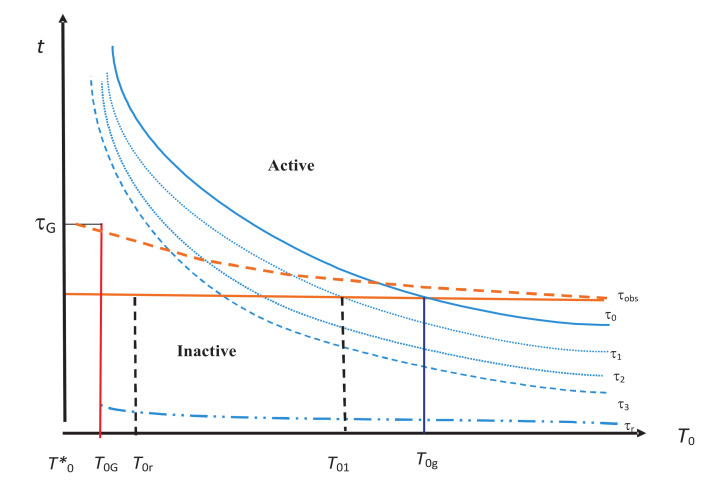
Schematic form of relaxation times $\left\{\tau_{n}\right\}$ as a function of the temperature $T_{0}$ for a fixed pressure $P_{0}$ of the medium. This figure will play an important role in the discussion of vitrification later. At low enough temperatures near $T_{0}^{*}<T_{0G}$, relaxation times become extremely large so that there is practically no relaxation over a long period of time. However, at $T_{0}>T_{0g}$, all internal variables have equilibrated over $\tau_{\mathrm{obs}}$ in the figure. We have drawn $\tau_{\mathrm{obs}}$ as a red solid horizontal line when it does not change and as a red broken line when it increases, as $T_{0}$ is reduced.

We find it very useful in this work to divide all internal variables in ξ into non-overlapping groups $\xi_{n}$ indexed by n=1,2,…, such that all internal variables in $\xi_{n}$ have the same relaxation time $\tau_{n}$ so that they equilibrate and are no longer independent of X for $\Delta t≳\tau_{n}$, and that all groups have distinct relaxation times ($\tau_{i}\neq \tau_{j}$ for i≠j). We supplement ξ by introducing a new group $\xi_{0}=X$ with relaxation time $\tau_{0}=\tau_{\mathrm{eq}}$ in order to compactify our notation so that $Z={\left\{\xi_{k}\right\}}_{k\geq 0}$. We also introduce the concept of hierarchy of relaxation times $\tau_{0}>\tau_{1}>\tau_{2}>\ldots$ associated with $\xi_{0},\xi_{1},\xi_{2},\ldots$, and state spaces $S_{0}\subset S_{1}\subset S_{2}\subset \ldots$, where $S_{n},n=0,1,2,\ldots$, is spanned by all $\xi_{k},k\leq n$, with relaxation times $\tau_{k}>\tau_{n+1}$. Physically, the hierarchy of relaxation times means that the longest relaxation time in $S_{n}$ is $\tau_{0}$ corresponding to $\xi_{0}=X$, and the shortest relaxation time is $\tau_{n}$ corresponding to $\xi_{n}$. Thus, if $\tau_{n}>\tau_{\mathrm{obs}}$, any $\xi_{k},k>n$, with relaxation time shorter than $\tau_{n}$, has already equilibrated (i.e., is no longer independent of X) and does not have to be used to specify the NEQ state. Thus, $S_{n}$ is the state space needed to specify the NEQ state for $\tau_{n}>\tau_{\mathrm{obs}}$. However, as $T_{0}$ is changed, both $\left\{\tau_{n}\right\}$ and $\tau_{\mathrm{obs}}$ can change as shown in Figure 2. This then affects the choice of the required state space $S_{n}$. Thus, the hierarchy becomes a central concept in our analysis.

One of the most important sets of internal variables is that associated with the vibrational modes in the system. We denote it by $\xi_{v}$, and it seems to have the property that it is always inactive. This is shown by the lowest lying relaxation time curve corresponding to $\tau_{v}$ in Figure 2. This is because we expect these modes to always come to equilibrium with the medium for any reasonable $\tau_{\mathrm{obs}}$.

## 3. Generalized Nonequilibrium Thermodynamics in the Extended Space

We are mostly interested in disordered states of a system in this work. Any ordered state, if it exists, is taken out of consideration from the start. Thus, the state space $S_{X}$ only contains disordered states. For vitrification, states in $S_{X}$ refer to the (physical or hypothetical) EQ states of the supercooled liquid. Defining such a restricted form of the equilibrium state space is very common in theoretical physics. For example, when we talk about an equilibrium crystal of a material, it is also defined in a restricted sense in which its molecules are not supposed to dissociate into constituent atoms. From now on, we will denote EQ quantities either by a subscript “eq” or “SCL” and NEQ quantities without any subscript. If we are interested in a ordered state, we will use a subscript “CR” to denote its quantity.

### 3.1. Equilibrium State

In EQ thermodynamics, a body is specified by a set X formed by its independent extensive observables (E,V,N, etc.); the set also serves the purpose of specifying the thermodynamic state (also known as the macrostate) M of the system. All EQ states belong to the state space $S_{X}$ as said above. The thermodynamic entropy of the body in equilibrium is a state function of X and is written as $S_{\mathrm{eq}}\left(X\right)$. It is one of the state functions of the system and is supposed to be differentiable except possibly at phase transitions, which we will not consider in this review. It satisfies the Gibbs fundamental relation:

(9) $$dS_{\mathrm{eq}}\left(X\right)=\left(dE+P_{0}dV-\mu_{0}dN+\ldots \right)/T_{0},$$

where we have shown only the terms related to E,V and *N*. The missing terms refer to the remaining variables in $X\equiv \left\{X_{p}\right\}$, and $T_{0},P_{0},\mu_{0},$ etc., have their standard meaning in equilibrium:

(10) $$\partial S_{\mathrm{eq}}/\partial E≐1/T_{0},\partial S_{\mathrm{eq}}/\partial V≐P_{0}/T_{0},\partial S_{\mathrm{eq}}/\partial N≐-\mu_{0}/T_{0},\ldots .$$

We have used a subscript 0 since in equilibrium, the fields of Σ and $\tilde{\Sigma }$ are the same.

### 3.2. Nonequilibrium States and Internal Equilibrium States

The above conclusion is most certainly not valid for a body out of equilibrium. If the body is not in equilibrium with its medium, its (macro)state M(t) will continuously change (relax), which is reflected in the changes in all of its physical quantities q(t) with time. Such variations mean that the states no longer belong to $S_{X}$. These states belong to the enlarged state space $S_{Z}$ spanned by Z=(X,ξ). The set ξ of internal variables [14,15,16,17,18,19,20,21,22,23] cannot be controlled from the outside [13]; a readable history of internal variables is available in a recent paper by Maugin [56]. They are used to characterize internal structures or inhomogeneity [16,19,21,22,23,24,25,57,58,59,60] in the system and are independent of the observables in X away from equilibrium, but become dependent on X in equilibrium. From Theorem 4 in [25], it follows that with a proper choice of the number of internal variables, the entropy can be written as S(Z(t)) with no explicit *t*-dependence. The situation is now almost identical to that of a body in equilibrium: the entropy is a function of Z(t) with no explicit time-dependence. This allows us to identify Z(t) as the set of NEQ state variables. States for which the entropy *S* becomes a state function of the state variable Z are called internal equilibrium (IEQ) states [18,22,24,25,57,58,59,60], and we write:

$$S_{\mathrm{ieq}}\left(t\right)=S\left(Z\left(t\right)\right)$$

for their entropy. This allows us to extend Equation (9) to:

(11) $$dS_{\mathrm{ieq}}\left(t\right)=\sum_{p}\left(\partial S_{\mathrm{ieq}}\left(t\right)/\partial Z_{p}\left(t\right)\right)dZ_{p}\left(t\right)$$

in which the partial derivatives are related to the fields of the system:

(12) $$\frac{\partial S_{\mathrm{ieq}}\left(t\right)}{\partial E(t)}≐\frac{1}{T(t)},\frac{\partial S_{\mathrm{ieq}}\left(t\right)}{\partial V(t)}≐\frac{P(t)}{T(t)},\frac{\partial S_{\mathrm{ieq}}\left(t\right)}{\partial N(t)}≐-\frac{\mu (t)}{T(t)},\ldots ,\frac{\partial S_{\mathrm{ieq}}\left(t\right)}{\partial \xi (t)}≐\frac{A(t)}{T(t)};$$

These fields will change in time unless the system has reached equilibrium. It is customary to call A the affinity [61]. For a fixed Z, $S_{\mathrm{ieq}}$ does not change in time. Hence, it must have the maximum possible value for fixed Z [52,53]. The EQ value of A vanishes [19,21]:

(13) $$A_{\mathrm{eq}}=0.$$

In this case, $S_{\mathrm{ieq}}$ is no longer a function of ξ, which means that ξ is no longer independent of X.

We consider the extension of the derivation given earlier [24] for the entropy of $\Sigma_{0}$ by including the internal variable contribution to obtain as the statement of the second law:

(14) $$\frac{dS_{0}\left(t\right)}{dt}=\left(\frac{1}{T(t)}-\frac{1}{T_{0}}\right)\frac{dE(t)}{dt}+\left(\frac{P(t)}{T(t)}-\frac{P_{0}}{T_{0}}\right)\frac{dV(t)}{dt}+\frac{A(t)}{T(t)}\cdot \frac{d\xi (t)}{dt}>0;$$

for an NEQ state. As the entropy of an isolated system $\Sigma_{0}$ can only increase, $dS_{0}\left(t\right)/dt$ cannot be negative, which explains the last inequality above for an NEQ process. The strict inequality will be replaced by an equality for $\Sigma_{0}$ in equilibrium. Each term in the first equation must be positive in accordance with the second law for an NEQ state.

It follows from Equation (30) that the above discussion also applies to an interacting system in a medium for which $d_{i}S/dt$ is nonnegative. Thus, we can apply it to a vitrification process in which the energy decreases with time during isothermal (fixed $T_{0}$ of $\Sigma_{0}$) relaxation. We must, therefore, have:

(15) $$T\left(t\right)>T_{0}$$

during any relaxation (at a fixed temperature and pressure of the medium) so that T(t) approaches $T_{0}$ from above [T(t)→$T_{0}^{+}$] and becomes equal to $T_{0}$ as the relaxation ceases and the equilibrium is achieved; the plus symbol is to indicate that the T(t) reaches $T_{0}$ from above.

The relaxation times for different internal variables in ξ depend on their nature and do not have to be the same. Indeed, the spectrum of relaxation times in various contexts such as in crystalline solids [62] and glasses [44] is intimately related to the existence of internal variables. Therefore, the spectrum of relaxation times will be pivotal in our discussion and will be picked up again in Section 4.

By attempting to describe NEQ properties of a system by invoking internal variables, one is able to explain a broad spectrum of NEQ phenomena, but it should be stated here that the choice and the number of state variables included in X or Z is not so trivial and must be determined by the nature of the experiments [22]. As we will see in Section 4, the observation time $\tau_{\mathrm{obs}}$ plays a central role in determining the relevant state variables during an experiment:

**Proposition** **1.** The state variables that determine the generalized NEQ thermodynamics are those whose relaxation times are longer than $\tau_{\mathrm{obs}}$.

**Proof.**  The proposition will be justified in Section 4; see the paragraph containing Equation (19). ☐

We will assume here that Z has been specified. For any IEQ states $M_{\mathrm{ieq}}\left(Z\right)$, we have $\tau_{\mathrm{ieq}}≲\tau_{\mathrm{obs}}<\tau_{\mathrm{eq}}$, where we have introduced the internal equilibration time $\tau_{\mathrm{ieq}}$ required for the system to come to an IEQ state in $S_{Z}$. As expected, $\tau_{\mathrm{ieq}}=\tau_{\mathrm{ieq}}\left(Z\right)$ depends on Z, but we will not explicitly exhibit its state dependence unless clarity is needed. These states appear for $\tau_{\mathrm{obs}}\geq \tau_{\mathrm{ieq}}$. There are many other states in $S_{Z}$ having non-state entropies that appear for $\tau_{\mathrm{obs}}<\tau_{\mathrm{ieq}}$. As $\tau_{\mathrm{obs}}\to \tau_{\mathrm{ieq}}$, we obtain an IEQ state $M_{\mathrm{ieq}}\left(Z\right)$. Therefore, there appears a delicate balance between $\tau_{\mathrm{obs}}$ and what internal variables we can describe by our thermodynamic approach using the concept of IEQ states. This leads us to consider the hierarchy of relaxation times, which is taken in Section 4.

It may appear to the reader that the concept of entropy being a state function is very restrictive. This is not the case, as this concept, although not recognized by several works, is implicit in the literature where the relationship of the thermodynamic entropy with state variables is investigated. To appreciate this, we observe that the entropy of a body in internal equilibrium [24,25] is given by the Boltzmann formula:

(16) S(Z(t))=lnW(Z(t)),

in terms of the number of microstates W(Z(t)) corresponding to Z(t). In classical NEQ thermodynamics [19], the entropy is always taken to be a state function. In the Edwards approach [63] for granular materials, all microstates are equally probable as is required for the above Boltzmann formula. Bouchbinder and Langer [58] assume that the NEQ entropy is given by Equation (16). Lebowitz [28,29] also takes the above formulation for his definition of the NEQ entropy. As a matter of fact, we are not aware of any work dealing with entropy computation that does not assume the NEQ entropy to be a state function. This does not, of course, mean that all states of a system are IEQ states. For states that are not in internal equilibrium, the entropy is not a state function so that it will have an explicit time dependence. However, as shown elsewhere [25], this can be avoided by enlarging the space of internal variables. The choice of how many internal variables are needed will depend on experimental time scales.

## 4. Hierarchy among Relaxation Times and Enlarged State Spaces

We now classify state variables in a hierarchical manner as below. In IEQ states, ξ has had enough time $\tau_{\mathrm{obs}}=\tau_{\mathrm{ieq}}<\tau_{\mathrm{eq}}$ for $M_{\mathrm{ieq}}$ to emerge. However, for $\tau_{\mathrm{obs}}<\tau_{\mathrm{ieq}}$, the states in $S_{Z}$ have not had enough time for $M_{\mathrm{ieq}}$ to emerge so that their entropy is a non-state function, which will continue to increase if the system is left isolated until it reaches $S_{\mathrm{ieq}}\left(X,\xi \right)$ and becomes a state function. The affinity A corresponding to ξ is nonzero in $M_{\mathrm{ieq}}$. If there were other internal variables $\xi^{\prime },\xi^{\prime \prime },\xi^{\prime \prime \prime },\ldots$ in the system, with relaxation times $\tau^{\prime },\tau^{\prime \prime },\tau^{\prime \prime \prime },\ldots$, respectively, that are distinct from ξ, then these must have equilibrated during $\tau_{\mathrm{ieq}}$ so that their affinities $A^{\prime },A^{\prime \prime },A^{\prime \prime \prime },\ldots$ have vanished, implying that they are no longer independent of X ($A^{\prime }=\partial S/\partial \xi^{\prime }=0$). This means that the entropy does not depend on them. It is clear that $\tau_{\mathrm{ieq}}$ forms an upper bound for the relaxation times $\tau^{\prime },\tau^{\prime \prime },\tau^{\prime \prime \prime },\ldots$. Thus, they play no role in $S_{Z}$. When the process is carried out somewhat faster ($\tau_{\mathrm{obs}}<\tau_{\mathrm{eq}}$) than that required for obtaining $M_{\mathrm{eq}}\left(X\right)$, then ξ has not had enough time to “equilibrate”, as we have discussed earlier [52,53] and A≠0.

Even if *S* does not depend on $\xi^{\prime },\xi^{\prime \prime },\xi^{\prime \prime \prime },\ldots$, we will see in Section 8 that they affect the thermodynamics of the system indirectly, a fact that does not seem to have been appreciated. For the moment, we will not consider the internal variables $\xi^{\prime },\xi^{\prime \prime },\xi^{\prime \prime \prime },\ldots$. We will consider them later and will denote them collectively by $\xi_{E}=\left(\xi^{\prime },\xi^{\prime \prime \prime },\xi^{\prime \prime \prime },\ldots \right)$.

The discussion below is somewhat abstract and intricate and requires patience on the part of the reader. The set-theoretic notation is perfectly suited for the abstract nature of the discussion. Some readers may find the set-theoretic notation cumbersome, but this is the price we must pay to make the discussion comprehensive, but compact.

To simplify our discussion, we assume that all internal variables in ξ are divided into non-overlapping groups $\xi_{n}$ indexed by n=1,2,…. We further assume that all internal variables in $\xi_{n}$ have the same relaxation time $\tau_{n}$ so that they equilibrate and are no longer independent of X for $\Delta t≳\tau_{n}$. The relaxation times depend strongly on X. Let us also define $\xi_{0}=X$ in order to compactify our notation below. Because of this, we can include $\xi_{0}=X$ whenever we speak of internal variables from now on, unless clarity is needed. The groups $\xi_{n},n=0,1,2,\ldots$ are indexed by *n* so that $\tau_{n}$’s appear in a decreasing order (with $\tau_{0}=\tau_{\mathrm{eq}}$):

(17) $$\tau_{0}>\tau_{1}>\tau_{2}>\ldots .$$

The relaxation times form a discrete set and not a continuum for simplicity. It is important that the set $\left\{\xi_{k}\right\}$ has a finite though large number of elements for a physically sensible thermodynamic description of the system; having an enormous number of elements will make the description unnecessarily too complex and completely useless for thermodynamics.

We now introduce the sequence of state spaces $\left\{S_{n}\right\}$, where $S_{n},n=0,1,2,\ldots$, is spanned by the union:

$$\xi^{\left(n\right)}≐\cup_{k=1}^{n}\xi_{k},n\geq 1,$$

of all $\xi_{k},k\leq n$, with relaxation times $\tau_{k}>\tau_{n+1}$, with $\xi^{\left(0\right)}$ (not to be confused with $\xi_{0}=X$) denoting an empty set, so that:

$$Z_{n}≐\left(X,\xi_{1},\xi_{2},\ldots ,\xi_{n}\right)\equiv \left(\xi_{0},\xi^{\left(n\right)}\right),n\geq 0.$$

Thus, $S_{0}=S_{X}$, formed by $Z_{0}=\xi_{0}=X$, is relevant when $\tau_{0}>\tau_{\mathrm{obs}}>\tau_{1}$. Similarly, $S_{1}$, formed by $Z_{1}=\left(\xi_{0},\xi^{\left(1\right)}\right)=\left(X,\xi_{1}\right),$ is relevant when $\tau_{1}>\tau_{\mathrm{obs}}>\tau_{2}$, and so on.

It is clear from the construction that the state spaces $S_{n},n=0,1,2,\ldots$, are ordered with increasing dimensions:

(18) $$S_{0}\subset S_{1}\subset S_{2}\subset \ldots .$$

The longest relaxation time in $S_{n}$ is $\tau_{0}$ corresponding to $\xi_{0}=X$ and the shortest relaxation time is $\tau_{n}$ corresponding to $\xi_{n}$. Any $\xi_{k},k>n$ with relaxation time shorter than $\tau_{n}$ need not be considered as it has already equilibrated and does not affect any state in $S_{n}$. We can summarize this conclusion as follows:

**Proposition** **2.** The additional internal variable $\xi_{k}$ in $S_{k}$ relative to $S_{k-1}$ equilibrates and plays no role (i.e., is absent) in any smaller state spaces $S_{l},l\leq k-1$, but participates in all state spaces $S_{l}$ larger than $S_{k-1}$, i.e., l≥k.

**Proof.**  See the discussion above. ☐

Let us consider some observation time $\tau_{\mathrm{obs}}$ used to observe a state M of an interacting system. We can always find a pair of neighboring state spaces $S_{n+1}⊃S_{n},n\geq 0$ satisfying:

(19) $$\tau_{n+1}<\tau_{\mathrm{obs}}<\tau_{n};$$

The two sides define a window $\Delta t_{n}≐\tau_{n}-\tau_{n+1}$ in which $\tau_{\mathrm{obs}}$ must lie. As $\tau_{\mathrm{obs}}>\tau_{n+1}$, no $\xi_{k}$’s, k>n, have to be considered to describe the state M as they have already equilibrated (cf. the discussion of $\overset{`}{\xi }$ above); thus, $S_{k},k>n$, play no role in describing M. As $\tau_{\mathrm{obs}}<\tau_{n}$, we need to consider all $\xi_{k},k\leq n$ to describe M. We must, therefore, use $S_{n}$ to describe M for a $\tau_{\mathrm{obs}}$ in this window; we denote M by $M(Z_{n})$ for clarity in this section. Among all the states in $S_{n}$, there are IEQ states $M_{\mathrm{ieq}}\left(Z_{n}\right)$ for which $S=S_{\mathrm{ieq}}\left(Z_{n}\right)$. This happens when $\tau_{\mathrm{obs}}≃\tau_{\mathrm{ieq}}\left(Z_{n}\right)\leq \tau_{n}$, $\tau_{\mathrm{ieq}}\left(Z_{n}\right)$ denoting the time required for $M(Z_{n})$ to evolve into $M_{\mathrm{ieq}}\left(Z_{n}\right)$; we will also use $\tau_{\mathrm{ieq}}^{\left(n\right)}$ or simply $\tau_{\mathrm{ieq}}$ to denote $\tau_{\mathrm{ieq}}\left(Z_{n}\right)$ in $S_{n}$ if no confusion will arise. For n=0, $\tau_{\mathrm{ieq}}\left(Z_{0}\right)$ simply refers to $\tau_{\mathrm{eq}}$.

There exists IEQ states $M_{\mathrm{ieq}}\left(Z_{n}\right)$ in $S_{n}$ for which $\xi_{n}$ is no longer independent of X; for these states, $\tau_{\mathrm{obs}}≃\tau_{\mathrm{ieq}}\left(Z_{n-1},\xi_{n}\left(X\right)\right)\equiv \tau_{\mathrm{ieq}}\left(Z_{n-1}\right)$. However, $\xi_{n}\to \xi_{n}\left(X\right)$ as $t\to \tau_{n}$ even if $M(Z_{n-1})$ in $S_{n-1}$ has not turned into $M_{\mathrm{ieq}}\left(Z_{n-1}\right)$. As achieving internal equilibrium will take some additional time, we have $\tau_{\mathrm{ieq}}\left(Z_{n-1}\right)>\tau_{n}$. We thus conclude that (with $\tau_{\mathrm{ieq}}^{\left(0\right)}$ representing $\tau_{\mathrm{eq}}$):

(20) $$\tau_{\mathrm{ieq}}^{\left(n\right)}<\tau_{\mathrm{ieq}}^{\left(n-1\right)},n>0,$$

which will be assumed in this work.

We now consider the window:

(21) $$\tau_{1}<\tau_{\mathrm{obs}}<\tau_{0}.$$

As $\tau_{0}>\tau_{\mathrm{obs}}>\tau_{1}$, $\xi_{1}$ has already equilibrated, so it need not be considered, $\xi_{0}=X$ has not yet equilibrated. Thus, the entropy must be a function only of the observables X, which we must write as $S_{\mathrm{ieq}}\left(X\left(t\right)\right)$, as it continues to vary. As $\tau_{\mathrm{obs}}\to \tau_{0}$, $X\left(t\right)\to X_{\mathrm{eq}}$, $S_{\mathrm{ieq}}$ continues to increase until it finally reaches $S_{\mathrm{eq}}$; there is no explicit time dependence as all $\xi_{k}$’s, k>0, have equilibrated; see also Landau and Lifshitz [7] and Wilks [10], where NEQ states with respect to the medium are treated as IEQ states in $S_{X}$. This is the most common way NEQ states in the literature are treated when internal variables are not invoked. This is only possible when $\tau_{\mathrm{obs}}$ satisfies Equation (21).

We now consider the remaining case:

(22) $$\tau_{\mathrm{obs}}\geq \tau_{0}.$$

This situation corresponds to the quasistatic case so that even $\xi_{0}=X$ has equilibrated to $X_{\mathrm{eq}}$, and we are dealing with an EQ state:

$$S=S_{\mathrm{eq}}=S\left(X_{\mathrm{eq}}\right).$$

We know that $\left\{\tau_{n}\right\}$ depend on the state of the system. In vitrification, which is of our primary interest here, they depend on the temperature $T_{0}$. It is commonly believed that $\tau_{n}$’s increase with decreasing $T_{0}$ as shown in Figure 2, where we show them as a function of $T_{0}$. From this figure, we observe that for a given $\tau_{\mathrm{obs}}$, drawn as a solid or broken line in red, $\xi_{E}$ correspond to the internal variables that lie in the inactive zone lying below $\tau_{\mathrm{obs}}$ (recall that $\xi_{0}=X$ is now included in internal variables). They have all equilibrated. The active zone corresponds to internal variables that lie above $\tau_{\mathrm{obs}}$. They have not equilibrated. For higher temperatures ($T_{0}>T_{0g}$), all internal variables are inactive. At lower temperatures, some of them become active and make the system out of equilibrium. At very low temperatures, all internal variables become active for their NEQ role. We will discuss this figure further in Section 6.

## 5. General Consideration

We have in Section 4 that for a given $\tau_{\mathrm{obs}}$, we can find the window $\Delta t_{n}$ satisfying Equation (21), which then determines the state space $S_{n}$ to describe any state M for the given $\tau_{\mathrm{obs}}$. The internal variables $\xi_{k}$’s, k>n, do not have to be considered as their affinities $A_{k}$’s have vanished for the given $\tau_{\mathrm{obs}}$. However, the situation is somewhat complicated for the following reason. As $\tau_{k}$’s are determined by time-dependent $Z_{n}$, the window will continue to change with time for a given $\tau_{\mathrm{obs}}$, so the value of *n* will have to adjusted as $\tau_{k}$’s change. The most simple solution for this complication is to allow considering all the internal variables regardless of whether they have equilibrated or not. The fact that A=0 for equilibrated internal variables means that their contribution to $d_{i}W$ will vanish so they will not affect the Gibbs fundamental relation. Despite this, as we will see later in Section 8, these internal variables leave their mark in relaxation. Therefore, from now on, we will consider the entire set Z in the thermodynamic approach.

### 5.1. First Law

The infinitesimal heat exchange between the medium $\tilde{\Sigma }$ and the system Σ will be denoted by $d_{e}Q\left(t\right)$; similarly, the infinitesimal work done on Σ by $\tilde{\Sigma }$ will be denoted by $d_{e}W\left(t\right)$. The subscript “e” is a reminder of the exchange. Then, the first law of thermodynamics is written as:

(23) $$dE\left(t\right)\equiv d_{e}Q\left(t\right)-d_{e}W\left(t\right)$$

in terms of exchange heat and work $d_{e}Q\left(t\right)=T_{0}d_{e}S\left(t\right)$ and $d_{e}W\left(t\right)=P_{0}dV\left(t\right)$, respectively; see Section 2. If there are other kinds of exchange work such as due to a magnetic field, an exchange of particles, etc., they can be subsumed in $d_{e}W\left(t\right)$. However, for simplicity, we will assume only the pressure-volume work in this work. Both quantities are controlled from outside the system. If the pressure P(t) of the system is different from the external pressure $P_{0}$ of the medium, then their difference gives rise to the internal work $d_{i}^{V}W\left(t\right)≐\left(P\left(t\right)-P_{0}\right)dV\left(t\right)$, which is dissipated within the system; we have added a superscript as a reminder that this particular internal work is due to volume variation. If there are internal variables, they do not contribute to $d_{e}W\left(t\right)$ as the corresponding EQ affinity $A_{0}=0$. Despite this, the internal variable ξ does internal work given by $d_{i}^{\xi }W\left(t\right)≐A\left(t\right)\cdot d\xi \left(t\right)$ and must be added to the internal work due to pressure difference. We thus identify the internal work $d_{i}W\left(t\right)$ as:

(24) $$d_{i}W\left(t\right)≐\left(P\left(t\right)-P_{0}\right)dV\left(t\right)+A\left(t\right)\cdot d\xi \left(t\right),$$

and the net work is:

(25) $$dW\left(t\right)=d_{e}W\left(t\right)+d_{i}W\left(t\right)=P\left(t\right)dV\left(t\right)+A\left(t\right)\cdot d\xi \left(t\right),$$

a quantity that depends only on Σ and is oblivious to the properties of $\;\tilde{\Sigma }$. Such a quantity is called a system-intrinsic (SI) quantity. Introducing a new quantity [25,53]:

(26) $$d_{i}Q\left(t\right)\equiv d_{i}W\left(t\right),$$

and the net heat:

(27) $$dQ\left(t\right)≐d_{e}Q\left(t\right)+d_{i}Q\left(t\right),$$

we can write the first law as:

(28) dE(t)=dQ(t)−dW(t).

As dE(t) and dW(t) are both SI-quantities, dQ(t) must also be a SI-quantity. Thus, the above formulation of the first law is in terms of quantities that refer to the system. There are no quantities that refer to $\tilde{\Sigma }$. We will call dQ(t) and dW(t) as the generalized heat dQ(t) added to and the generalized work dW(t) done by the system [24,25]. We will reserve exchange heat and work for $d_{e}Q\left(t\right)=T_{0}d_{e}S\left(t\right)$ and $d_{e}W\left(t\right)=P_{0}dV\left(t\right)$, respectively, throughout this work; see Section 2. Remembering this, we will also call generalized heat and work as simply heat and work, respectively, for brevity.

### 5.2. Second Law

The second law states that the irreversible (denoted by a suffix i) entropy $d_{i}S$ generated in any infinitesimal physical process going on within a system satisfies the inequality:

(29) $$d_{i}S\geq 0;$$

the equality occurs for a reversible process. For the isolated system $\Sigma_{0}$, we must have (see Equation (3)):

(30) $$dS_{0}=d_{i}S_{0}=d_{i}S\geq 0.$$

As the thermodynamic entropy is not measurable except when the process is reversible, the second law remains useless as a computational tool. In particular, it says nothing about the rate at which the irreversible entropy increases. Therefore, it is useful to obtain a computational formulation of the entropy, the statistical entropy. This will be done in the next section. The onus is on us to demonstrate that the statistical entropy also satisfies this law if it is to represent the thermodynamic entropy. This by itself does not prove that the two are the same. It has not been possible to show that the statistical entropy is identical to the thermodynamic entropy in general. Here, we show their equivalence only when the NEQ thermodynamic entropy is a state function of NEQ state variables to be introduced below.

### 5.3. Internal Equilibrium Thermodynamic

For a body in internal equilibrium, its entropy *S* is a function of E,V and ξ. Introducing the corresponding fields:

(31) $$\left(\partial S/\partial E\right)=\beta \left(t\right)≐1/T\left(t\right),\left(\partial S/\partial V\right)=\beta \left(t\right)P\left(t\right),\left(\partial S/\partial \xi \right)=\beta \left(t\right)A\left(t\right),$$

we can write down the differential:

dS(t)=β(t)[dE(t)+P(t)dV(t)+A(t)·dξ(t)],

which can be inverted to express dE(t) as follows:

(32) dE(t)=T(t)dS(t)−P(t)dV(t)−A(t)·dξ(t).

Comparing with Equation (28), we conclude an identity:

(33) dQ(t)=T(t)dS(t),

regardless of the number of internal variables used to describe Σ.

We now write $dQ=T_{0}d_{e}S\left(t\right)+T_{0}d_{i}S\left(t\right)+\left[T\left(t\right)-T_{0}\right]dS\left(t\right)=d_{e}Q\left(t\right)+T_{0}d_{i}S\left(t\right)+\left[T\left(t\right)-T_{0}\right]dS\left(t\right)$. From this and using Equation (26), we conclude that:

(34) $$d_{i}Q\left(t\right)=T_{0}d_{i}S\left(t\right)+\left[T\left(t\right)-T_{0}\right]dS\left(t\right)=Td_{i}S\left(t\right)+\left[T\left(t\right)-T_{0}\right]d_{e}S\left(t\right)=d_{i}W\left(t\right),$$

which can be used to express $d_{i}S\left(t\right)$ as follows:

(35) $$\begin{array}{cc} T_{0}d_{i}S\left(t\right) & =\left[T_{0}-T\left(t\right)\right]dS\left(t\right)+\left[P\left(t\right)-P_{0}\right]dV\left(t\right)+A\left(t\right)\cdot d\xi \left(t\right); \end{array}$$

(36) $$\begin{array}{cc} Td_{i}S\left(t\right) & =\left[T_{0}-T\left(t\right)\right]d_{e}S\left(t\right)+\left[P\left(t\right)-P_{0}\right]dV\left(t\right)+A\left(t\right)\cdot d\xi \left(t\right). \end{array}$$

Since $dS\left(t\right),d_{e}S\left(t\right),dV\left(t\right)$ and dξ(t) are independent variations, each of the three contributions on the right side in each equation must be non-negative:

(37a) $$\begin{array}{cc} {}[T_{0}-T\left(t\right)]dS\left(t\right) & \geq 0, \end{array}$$

(37b) $$\begin{array}{cc} {}[T_{0}-T\left(t\right)]d_{e}S\left(t\right) & \geq 0, \end{array}$$

(37c) $$\begin{array}{cc} {}[P\left(t\right)-P_{0}]dV\left(t\right) & \geq 0, \end{array}$$

(37d) $$\begin{array}{cc} A(t)\cdot d\xi (t) & \geq 0, \end{array}$$

to comply with the second law requirement $d_{i}S\left(t\right)\geq 0$; we are assuming $T_{0}$ and T(t) are positive. The factors $T_{0}-T\left(t\right),$ $P\left(t\right)-P_{0}$ and A(t) in front of the extensive variations are the corresponding thermodynamic forces that act to bring the system to equilibrium. In the process, each force has its own irreversible entropy generation [25]. The last inequality implies that each independent component $\xi_{k}\in \xi$ must satisfy $A_{k}\left(t\right)d\xi_{k}\left(t\right)\geq 0$. There will be no irreversible entropy generation, and the equalities occur when thermodynamic forces vanish, which is the situation for a reversible process.

It should be noted that Equation (37b) simply states that heat exchanges (flows) from hot to cold. To see this, we use the equality $d_{e}S\left(t\right)=d_{e}Q\left(t\right)/T_{0}$ to rewrite the equation as $\left[T_{0}-T\left(t\right)\right]d_{e}Q\left(t\right)\geq 0$. If $T_{0}>T\left(t\right)$, heat is exchanged to the system; if $T_{0}<T\left(t\right)$, heat is exchanged from the system.

It follows from the last two inequalities in Equation (37) that:

(38) $$d_{i}W\left(t\right)\geq 0.$$

This means that $d_{i}W\left(t\right)$ truly represents irreversibility or dissipation within the system. We note that while each term in $d_{i}W\left(t\right)$ is non-negative, this is not so for $d_{i}Q\left(t\right)$ written in the form:

(39a) $$\begin{array}{cc} d_{i}Q\left(t\right) & =T_{0}d_{i}S\left(t\right)+\left[T\left(t\right)-T_{0}\right]dS\left(t\right), \end{array}$$

(39b) $$\begin{array}{c} =T\left(t\right)d_{i}S\left(t\right)+\left[T\left(t\right)-T_{0}\right]d_{e}S\left(t\right) \end{array}$$

in which the first term is non-negative, but the second term is non-positive. This not only means that the physics of $d_{i}Q\left(t\right)$ and $d_{i}S\left(t\right)$ is very different, but also that:

(40) $$d_{i}Q\left(t\right)\leq T_{0}d_{i}S\left(t\right)\;,dQ\left(t\right)\leq T_{0}dS\left(t\right);$$

the equalities occur only for isothermal ($T=T_{0}$) or adiabatic (dS=0) processes.

Let us consider the Helmholtz free energy $H\left(S,V,\xi ,P_{0}\right)=E\left(S,V,\xi \right)+P_{0}V\left(t\right)$ [24,25] in terms of the external pressure $P_{0}$ of the medium. We can treat $HH(S,V,\xi ,P_{0})$ as an SI-quantity by treating $P_{0}$ as a parameter. It is easy to see that:

(41) $$dH=TdS\left(t\right)-\left[P\left(t\right)-P_{0}\right]dV\left(t\right)-A\left(t\right)\cdot d\xi \left(t\right)+V\left(t\right)dP_{0}.$$

The above differential clearly shows that the enthalpy *H* is a function of S,V,ξ and $P_{0}$. Recall that for an EQ state, $H(S,P_{0})$ is not a function of *V*, so it is a Legendre transform of E(S,V) with respect to V(t). In other words, ∂H/∂V=0. What we see from above is that, for an NEQ states, *H* is not a Legendre transform of *E* with respect to *V*. This is clearly seen by evaluating:

$$\partial H/\partial V=P\left(t\right)-P_{0}\neq 0,$$

as the pressure difference need not vanish in an irreversible process. Despite this, dH has no irreversible component as we easily find that:

(42) $$dH=TdS\left(t\right)-d_{i}W\left(t\right)+V\left(t\right)dP_{0}=d_{e}Q+V\left(t\right)dP_{0},$$

regardless of the number and nature of the internal variables; we have used here Equations (33) and (26). Thus, dH only contains exchange quantities as both terms on the right side are controllable from outside the system. As such, it does not have any spontaneous or irreversible relaxation. For an isobaric process, $dP_{0}=0$, so dH reduces to:

(43) $$dH=d_{e}Q.$$

The above equality, which is well known for a reversible process, remains valid no matter how irreversible a process is. Thus, it must remain valid for supercooled liquids and glasses. Observe that just as $d_{i}E=0$ (see Equation (6)), so is $d_{i}H=0$, with $d_{e}H=dH=d_{e}Q$.

Let us now consider the Gibbs free energy $G\left(S,V,\xi ,T_{0},P_{0}\right)≐E\left(S,V,\xi \right)-T_{0}S\left(t\right)+P_{0}V\left(t\right)$ [24,25] in terms of the external temperature $T_{0}$ and pressure $P_{0}$ of the medium. As is the case with the enthalpy, the Gibbs free energy is also not a Legendre transform of E(S,V,ξ) with respect to S(t) and V(t). We find that:

(44) $$dG=\left[T\left(t\right)-T_{0}\right]dS\left(t\right)-d_{i}W\left(t\right)-S\left(t\right)dT_{0}+V\left(t\right)dP_{0}=-T_{0}d_{i}S\left(t\right)-S\left(t\right)dT_{0}+V\left(t\right)dP_{0},$$

in which the first term can be identified as $d_{i}G≐-T_{0}d_{i}S\left(t\right)$ and the remainder as $d_{e}G≐-S\left(t\right)dT_{0}+V\left(t\right)dP_{0}$. At fixed $T_{0}$ and $P_{0}$, we have:

$$dG=d_{i}G=-T_{0}d_{i}S\left(t\right)\leq 0,$$

showing that the Gibbs free energy decreases during spontaneous relaxation such as on a glass.

## 6. Entropy Bound during Vitrification

**Figure 3:**
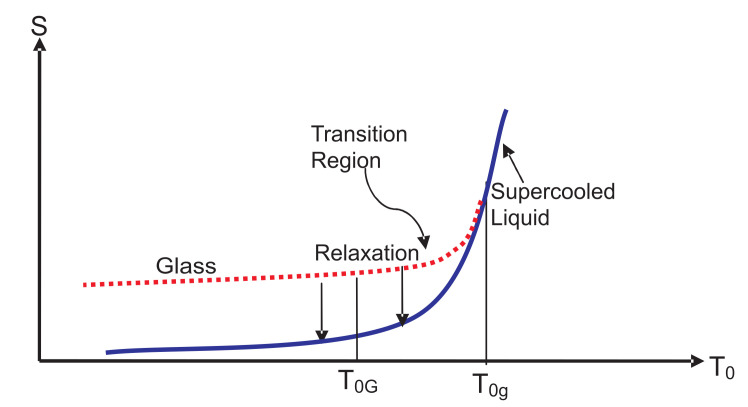
Schematic behavior of the entropy: equilibrated supercooled liquid (solid curve) and a glass (dotted curve) during vitrification as a function of the temperature $T_{0}$ of the medium. Structures appear to freeze at and below $T_{0G}$; see the text. The transition region between $T_{0g}$ and $T_{0G}$ over which the liquid turns into a glass has been exaggerated to highlight the point that the glass transition is not a sharp point. For all $T_{0}<T_{0g}$, the system undergoes isothermal (fixed $T_{0}$) structural relaxation in time towards the supercooled liquid shown by the downwards arrows. The entropy of the supercooled liquid is shown to extrapolate to zero, but that of the glass to a positive value $S_{R}$ at absolute zero, per our assumption.

We now apply the IEQ thermodynamics of the last section to the vitrification process, which is carried out at some cooling rate as follows. The discussion in this section is an elaboration and extension of our earlier discussion [53,64,65,66,67,68] and follows the approach first used by Bestul and Chang [48] and later by Sethna and coworkers [69,70]. The temperature of the medium is isobarically changed by some small, but fixed $\Delta T_{0}$ from the current value to the new value, and we wait for (not necessarily fixed) time $\tau_{\mathrm{obs}}$ at the new temperature to make an instantaneous measurement on the system before changing the temperature again. At some temperature $T_{0g}$, the relaxation time $\tau_{0}=\tau_{\mathrm{eq}}$, which continuously increases as the temperature is lowered (see Figure 2), becomes equal to $\tau_{\mathrm{obs}}$, as shown in Figure 3. The location of $T_{0g}$ depends on the rate of cooling, i.e., on $\tau_{\mathrm{obs}}$, which is clear from the figure. The crossing $T_{0g}$ is lower for the broken $\tau_{\mathrm{obs}}$ than for the solid $\tau_{\mathrm{obs}}$. There are several other crossings at $T_{01},T_{02},\ldots$ (see Figure 2), at which $\tau_{\mathrm{obs}}$ crosses other relaxation curves for $\tau_{1},\tau_{2},\ldots$, respectively. The crossing again depends on whether we take the solid or the broken curve for $\tau_{\mathrm{obs}}$. Let $T_{0R}>T_{0G}$ denote the temperature of the last such crossing (not shown in the figure) before $T_{0G}$. Just below $T_{0g}$, the structures are not yet frozen; they “freeze” at a lower temperature $T_{0G}$ (not too far from $T_{0g})$ to form an amorphous solid with a viscosity $\eta_{G}≃10^{13}$ poise corresponding to some time scale $t_{G}$; see Figure 3. This solid is identified as a glass determined by the choice of $\eta_{G}$ or $t_{G}$. At $T_{0G}$, the relaxation time $\tau_{R}$ is at least $\tau_{G}$. Over the glass transition region between $T_{0G}$ and $T_{0g}$ in Figure 3, the NEQ liquid gradually turns from an EQ supercooled liquid at or above $T_{0g}$ into a glass at or below $T_{0G}$, a picture already known since Tammann [2]; see also [71]. Over this region, some dynamical properties such as the viscosity vary continuously, but very rapidly. However, thermodynamic quantities such as the volume or the enthalpy change continuously, but slowly. As is evident from Figure 2, more and more internal variables become active as the temperature is reduced and will determine the thermodynamics in this region. Below $T_{0G}$, all of these are almost “frozen” except those in the inactive zone such as $\xi_{v}$ corresponding to the relaxation time $\tau_{v}$, representing localized oscillations within cells in the cell model [72]; see the discussion in Section 8 and Section 9.

As the observation time $\tau_{\mathrm{obs}}$ is increased, the equilibrated supercooled liquid continues to lower temperatures before the appearance of $T_{0g}$. In the hypothetical limit $\tau_{\mathrm{obs}}\to \infty$, it is believed that the equilibrated supercooled liquid will continue to lower temperatures without any interruption and is shown schematically by the solid blue curve in Figure 3. We overlook the possibility of the supercooled liquid ending in a spinodal that has been seen theoretically [73]. It is commonly believed that this entropy will vanish at absolute zero ($S_{\mathrm{SCL}}\left(0\right)\equiv 0$), as shown in the figure. As we are going to be interested in $S_{\mathrm{SCL}}\left(T_{0}\right)$ over $\left(0,T_{0g}\right)$, we must also acknowledge the possibility of an ideal glass transition in the system. If one believes in an ideal glass transition, then there would be a singularity in $S_{\mathrm{SCL}}\left(T_{0}\right)$ at some positive temperature $T_{K}<T_{0G},$ below which the system will turn into an ideal glass whose entropy will also vanish at absolute zero [34, see also the references cited there]. The possibility of an ideal glass transition, which has been discussed in a recent review elsewhere [34], will not be discussed further in this work. All that will be relevant in our discussion here is the fact that the entropy vanishes in both situations ($S_{\mathrm{SCL}}\left(0\right)\equiv 0$). However, it should be emphasized that the actual value of $S_{\mathrm{SCL}}\left(0\right)$ has no relevance for the theorems we derive below.

It is a common practice to think of the glass transition as occurring at a point that lies between $T_{0g}$ and $T_{0G}$. We have drawn entropy curves (glass and SCL) in Figure 3 for a process of vitrification in a cooling experiment. The entropy curves $S_{g}\left(T_{0},t\right)\;$ for glass emerges out of $S_{\mathrm{SCL}}\left(T_{0}\right)$ at $T_{0g}$ for a given $\tau_{\mathrm{obs}}$ in such a way that it lies above that of SCL for $T_{0g}>T_{0}\geq 0$. At any nonzero temperature $T_{0}$, $S(T_{0},t)$ approach $S_{\mathrm{SCL}}\left(T_{0}\right)$ from above during isothermal (fixed temperature of the medium) relaxation; see the two downward vertical arrows. These relaxations are discussed in the next section.

The concept of internal equilibrium is also a common practice now-a-days for glasses [2,4]. Employing the concept of internal equilibrium provides us with an instantaneous Gibbs fundamental relation (see Equation (32)), which determines instantaneous temperature, pressure, etc., of the system.

We now prove the entropy bounds:

(45) $$S_{R}\equiv S\left(0\right)>S_{\mathrm{expt}}\left(0\right)>S_{\mathrm{SCL}}\left(0\right).$$

in the form of Theorems 3 and 4. We will only consider isobaric cooling (we will not explicitly exhibit the pressure in this section), which is the most important situation for glasses. The process is carried out along some path from an initial state A at temperature $T_{0A}$ in the supercooled liquid state, which is still higher than $T_{0g}$, to the state A_0_ at absolute zero. The state A_0_ depends on the path A→A_0_, which is implicit in the following. The change dS between two neighboring points along such a path is [19,21,24,25,61] $dS=d_{e}S+d_{i}S$; for an NEQ system, the two parts of dS are path dependent. The component:

(46) $$d_{e}S\left(t\right)=-d_{e}Q\left(t\right)/T_{0}\equiv C_{P}dT_{0}/T_{0}$$

represents the reversible entropy exchange with the medium in terms of the heat $d_{e}Q\left(t\right)$ given out by the glass at time *t* to the medium whose temperature at that instant is $T_{0}$. The component $d_{i}S>0$ represents the irreversible entropy generation in the irreversible process; see Equation (29). In general, it contains, in addition to the contribution from the irreversible heat transfer with the medium, contributions from all sorts of viscous dissipation going on within the system and normally requires the use of internal variables [19,21,24,25,61]. The equality in Equation (29) holds for a reversible process, which we will no longer consider unless stated otherwise. The strict inequality $d_{i}S>0$ occurs only for an irreversible process such as in a glass.

**Theorem** **3.** *The experimentally-observed (extrapolated) non-zero entropy $S_{\mathrm{expt}}\left(0\right)$ at absolute zero in a vitrification process is a strict lower bound of the residual entropy of any system:*

$$S_{R}\equiv S\left(0\right)>S_{\mathrm{expt}}\left(0\right).$$

**Proof.**  We have along A→A_0_:

(47) $$S\left(0\right)=S\left(T_{0}\right)+\int_{A}^{A_{0}}d_{e}S+\int_{A}^{A_{0}}d_{i}S,$$

where we have assumed that there is no latent heat in the vitrification process. The first integral is easily determined experimentally since it is expressible in terms of the exchange heat:

$$\int_{A}^{A_{0}}d_{e}S=-\int_{A}^{A_{0}}\frac{d_{e}Q}{T_{0}}.$$

The second integral in (47) is always positive, but almost impossible to measure as it involves thermodynamic forces (see Equation (37a)):

(48) $$\int_{A}^{A_{0}}d_{i}S=\int_{A}^{A_{0}}\frac{\left[T_{0}-T\left(t\right)\right]dS\left(t\right)+\left[P\left(t\right)-P_{0}\right]dV\left(t\right)+A\left(t\right)\cdot d\xi \left(t\right)}{T_{0}}>0.$$

It involves knowing that since the residual entropy $S_{R}$ is, by definition, the entropy S(0) at absolute zero, we obtain the important result:

(49) $$S_{R}\equiv S\left(0\right)>S_{\mathrm{expt}}\left(0\right)≐S\left(T_{0A}\right)+\int_{T_{0A}}^{0}C_{P}dT_{0}/T_{0}.$$

This proves Theorem 3. ☐

The irreversibility during vitrification does not allow for the determination of the entropy exactly, because evaluating the integral in Equation (48) is not feasible [2,25]. The forward inequality:

$$S_{R}-S_{\mathrm{expt}}\left(0\right)=\int_{A}^{A_{0}}d_{i}S>0$$

is due to the irreversible entropy generation from all possible sources [19,21,24,25,61]. The inequality is made strict as we are treating the NEQ glass with $\tau_{\mathrm{obs}}<\tau_{\mathrm{eq}}\left(T_{0}\right)$ and clearly establishes that the residual entropy at absolute zero must be strictly larger than the “experimentally- or calorimetrically-measured” $S_{\mathrm{expt}}\left(0\right)$.

**Theorem** **4.** *The calorimetrically-measured (extrapolated) entropy during processes that occur when $\tau_{\mathrm{obs}}<\tau_{\mathrm{eq}}\left(T_{0}\right)$ for any $T_{0}<T_{0g}$ is larger than the hypothetical supercooled liquid entropy at absolutely zero:*

$$S_{\mathrm{expt}}\left(0\right)>S_{\mathrm{SCL}}\left(0\right).$$

**Proof.**  Let ${\dot{Q}}_{e}\left(t\right)\equiv d_{e}Q\left(t\right)/dt$ be the rate of net heat loss by the system during $\tau_{\mathrm{obs}}<\tau_{\mathrm{eq}}\left(T_{0}\right)$ as it relaxes isothermally at some fixed $T_{0}$. For each temperature interval $dT_{0}<0$ below $T_{0g}$, we have:

$$\left|d_{e}Q\right|\equiv C_{P}\left|dT_{0}\right|=\int_{0}^{\tau_{\mathrm{obs}}}\left|{\dot{Q}}_{e}\right|dt<{\left|d_{e}Q\right|}_{\mathrm{eq}}\left(T_{0}\right)≐\int_{0}^{\tau_{\mathrm{eq}}\left(T_{0}\right)}\left|{\dot{Q}}_{e}\right|dt,\;\;\;\;\;\;T_{0}<T_{0g}$$

where ${\left|d_{e}Q\right|}_{\mathrm{eq}}\left(T_{0}\right)>0$ denotes the net heat loss by the system to come to equilibrium, i.e., become supercooled liquid during cooling at $T_{0}$. For $T_{0}\geq T_{0g}$, $dQ\equiv dQ_{\mathrm{eq}}\left(T_{0}\right)≐C_{P,\mathrm{eq}}dT_{0}$. Thus, the entropy loss observed experimentally with $\tau_{\mathrm{obs}}<\tau_{\mathrm{eq}}\left(T_{0}\right)$ is less than the entropy loss if the system is allowed to come to SCL at each temperature $T_{0}$. We thus conclude that:

(50) $$S_{\mathrm{expt}}\left(0\right)>S_{\mathrm{SCL}}\left(0\right).$$

This proves Theorem 4. ☐

The strict inequality above is the result of the fact that glass is an NEQ state. Otherwise, we will have $S_{\mathrm{expt}}\left(0\right)\geq S_{\mathrm{SCL}}\left(0\right)$ for any arbitrary state.

The difference $S_{R}-$$S_{\mathrm{expt}}\left(0\right)$ would be larger the more irreversible the process is. The quantity $S_{\mathrm{expt}}\left(0\right)$ can be determined calorimetrically by performing a cooling experiment. We take $T_{0A}$ to be the melting temperature $T_{0M}$ and uniquely determine the entropy of the supercooled liquid at $T_{0M}$ by adding the entropy of melting to the crystal entropy $S_{\mathrm{CR}}\left(T_{0M}\right)$ at $T_{0M}$. The latter is obtained in a unique manner by integration along a reversible path from $T_{0}=0$ to $T_{0}=T_{0M}$:

$$S_{\mathrm{CR}}\left(T_{0M}\right)=S_{\mathrm{CR}}\left(0\right)+\int_{0}^{T_{0M}}C_{P,\mathrm{CR}}dT_{0}/T_{0};$$

here, $S_{\mathrm{CR}}\left(0\right)$ is the entropy of the crystal at absolute zero, which is traditionally taken to be zero in accordance with the third law, and $C_{P,\mathrm{CR}}\left(T_{0}\right)$ is the isobaric heat capacity of the crystal. This then uniquely determines the entropy of the liquid to be used in the right-hand side in Equation (49). We will assume that $S_{\mathrm{CR}}\left(0\right)=0$. Thus, an experimental determination of $S_{\mathrm{expt}}\left(0\right)$ is required to give the lower bound to the residual entropy in Equation (45). Experimental evidence for a non-zero value of $S_{\mathrm{expt}}\left(0\right)$ is abundant as discussed by several authors [35,44,45,46,74,75,76]; various textbooks [2,4] also discuss this issue. Goldstein [44] gives a value of $S_{R}≃15.1$ J/K mol for *o*-terphenyl from the value of its entropy at $T_{0}=2$ K. However, Equation (50) gives a mathematical justification of $S_{\mathrm{expt}}\left(0\right)>0$. The strict inequality proves immediately that the residual entropy cannot vanish for glasses, which justifies the curve “Glass” in Figure 3. The relevance of the residual entropy has been discussed by several authors in the literature [30,32,38,39,40,41,44,74,75,77].

By considering the state A_0_ above to be a state A_0_ of the glass in a medium at some arbitrary temperature $T_{0}^{\prime }$ below $T_{0g}$, we can get a generalization of Equation (49):

(51) $$S\left(T_{0}^{\prime }\right)>S_{\mathrm{expt}}\left(T_{0}^{\prime }\right)≐S\left(T_{0}\right)+\int_{T_{0}}^{T_{0}^{\prime }}C_{P}dT_{0}/T_{0}.$$

We again wish to remind the reader that all quantities depend on the path A→A_0_, which we have not exhibited. By replacing $T_{0}$ by the melting temperature $T_{0M}$ and $T_{0}^{\prime }$ by $T_{0}$, adding the entropy $\tilde{S}\left(T_{0M}\right)$ of the medium on both sides in the above inequality and rearranging terms, we obtain (with $S_{L}\left(T_{0M}\right)=S_{\mathrm{SCL}}\left(T_{0M}\right)$ for the liquid):

(52) $$S_{L}\left(T_{0M}\right)+\tilde{S}\left(T_{0M}\right)\leq S\left(T_{0}\right)+\tilde{S}\left(T_{0M}\right)-\int_{T_{0M}}^{T_{0}}C_{P}dT_{0}/T_{0},$$

where we have also included the equality for a reversible process. This provides us with an independent derivation of the inequality given by Sethna and coworkers [69,70].

It is also clear from the derivation of Equation (50) that the inequality can be generalized to any temperature $T_{0}<T_{0g}$ with the result:

(53) $$S_{\mathrm{expt}}\left(T_{0}\right)>S_{\mathrm{SCL}}\left(T_{0}\right),$$

with $S_{\mathrm{expt}}\left(T_{0}\right)\to S_{\mathrm{SCL}}\left(T_{0}\right)$ as $T_{0}\to T_{0g}$ from below.

While we have only demonstrated the forward inequality, the excess $S_{R}-S_{\mathrm{expt}}\left(0\right)$ can be computed in NEQ thermodynamics [19,21,24,25,61], which provides a clear prescription for calculating the irreversible entropy generation. The calculation will, of course, be system dependent and will require detailed information. Gutzow and Scmelzer [74] provide such a procedure with a single internal variable, but under the assumption of equal temperature and pressure for the glass and the medium. However, while they comment that $d_{i}S\geq 0$, whose evaluation requires system-dependent properties, their main interest is to only show that it is negligible compared to $d_{e}S$.

We have prove, Theorems 3 and 4 by considering only the system without paying any attention to the medium. For Theorem 3, we require the second law, i.e., Equation (29). This is also true of Equation (51). The proof of Theorem 4 requires the constraint $\tau_{\mathrm{obs}}<\tau_{\mathrm{eq}}\left(T_{0}\right)$ for any $T_{0}<T_{0g}$, which leads to an NEQ state. The same is also true of Equation (53).

We have focused on the system in this section. This does not mean that the conclusion would be any different had we brought the medium into our discussion. This is seen from the derivation of the inequality in Equation (52) from Equation (51).

## 7. Entropy and Enthalpy during Isothermal Relaxation

We wish to consider isothermal relaxation in an isobaric cooling experiment carried out at a fixed pressure $P_{0}$. Let us assume that Σ is in equilibrium at some temperature $T_{0}^{\prime }\leq T_{0g}$ of some medium ${\tilde{\Sigma }}^{\prime }$. We change to a different medium $\tilde{\Sigma }$ at $T_{0}<T_{0}^{\prime },P_{0}$ and bring Σ in its contact. Initially, the temperature $T\left(0\right)$ of Σ is $T\left(0\right)=T_{0}^{\prime }>T_{0}$, so it is out of equilibrium with the new medium, and its temperature T(t) will strive to get closer to $T_{0}$ as we wait for Σ to come to equilibrium with $\tilde{\Sigma }$; see Equation (15). The initial entropy $S\left(T_{0},0\right)=S_{\mathrm{SCL}}\left(T_{0}^{\prime }\right)>S_{\mathrm{SCL}}\left(T_{0}\right)$. If the system is now allowed to equilibrate, it will undergo spontaneous (isothermal) relaxation at fixed $T_{0}$ so that $S\left(T_{0},t\right)\to S_{\mathrm{SCL}}\left(T_{0}\right)$ in the time during which its temperature changes. We assume that the relaxation times of $\xi_{n}$ as a function of T(t) are similar to those shown in Figure 2; all we need to do is to replace $T_{0}$ by T(t). During relaxation, the entropy of the glass is supposed to decrease. This is what we expect intuitively as the arrows show in Figure 3. We now wish to consider such a relaxation and determine the behavior of thermodynamic functions such as the entropy, enthalpy, etc., using IEQ thermodynamics introduced above. We prove two additional theorems in this section. The theorems are general even though we have in mind NEQ states including glasses obtained under the condition $\tau_{\mathrm{obs}}<\tau_{\mathrm{eq}}\left(T_{0}\right)$ for any $T_{0}<T_{0g}$. We consider the system to be in internal equilibrium with temperature T(t), pressure P(t), etc. We remind the reader that all processes that go on within the medium occur at constant temperature $T_{0}$, pressure $P_{0}$, etc. Thus, there will not be any irreversible process going on within the medium. All irreversible processes will go on within the system.

We will exploit below the strict inequalities in Equation (37) to derive a bound on the rate of entropy variation. For a system out of equilibrium, the instantaneous entropy S(t) and volume V(t) seem to play the role [24] of “internal variables,” whose “affinities” are given by the corresponding thermodynamic forces $T_{0}-T\left(t\right)$ and $P\left(t\right)-P_{0}$, respectively. This fact is not commonly appreciated in the glass literature to the best of our knowledge. Even during an isobaric vitrification, there is no fundamental reason to assume that the pressure *P* of the system is always equal to the external pressure $P_{0}$. However, it is a common practice to assume the two to be the same, which may not be a poor approximation in most cases. We will not generally make such an approximation in this work.

We now state Theorem 5.

**Theorem** **5.** *The entropy of a glass reaches that of the supercooled liquid from above during relaxation at fixed $T_{0},P_{0}$ of the mediums. Thus,*

$$S>S_{\mathrm{SCL}},$$

*so that the entropy variation in time has a unique direction as shown by the downward arrows in Figure 3.*

**Proof.**  It follows from Equations (15) and (37a) that for any NEQ state during relaxation (fixed $T_{0},P_{0}$):

(54) dS(t)/dt<0;

the inequality turns into an equality once equilibrium is reached. In other words, during relaxation,

$$S\left(T_{0},P_{0},t\right)\to S_{\mathrm{SCL}}^{+}\left(T_{0},P_{0}\right);$$

the plus symbol is again to indicate that the glass entropy reaches $S_{\mathrm{SCL}}\left(T_{0},P_{0}\right)$ from above. This completes the proof of Theorem 5. ☐

We have shown $T_{0},P_{0}$ in $S\left(T_{0},P_{0},t\right)\equiv S\left(T\left(t\right),P\left(t\right),A\left(t\right)\right)$ to emphasize that the result is general during any relaxation. In the derivation, we have only used the second law. Being a general result, it should be valid for any real glass. Above $T_{0g}$, the system is always in equilibrium with the medium so its temperature is the same as $T_{0}$. Below $T_{0g}$, when the system is not in equilibrium with the medium, then $T\left(t\right)>T_{0}$ in accordance with Equation (15) based on the experimental observation. Any theory, such as the one proposed in [78,79,80,81] and known as the entropy loss view of the glass transition, in which $S(T_{0},P_{0},t)$ drops below $S_{\mathrm{SCL}}\left(T_{0},P_{0}\right)$, is such that:

(55) $$S\left(T_{0},t\right)\leq S_{\mathrm{SCL}}\left(T_{0}\right).$$

In this case, during relaxation, dS(t)>0, so that $(T_{0}-T\left(t\right))dS\left(t\right)<0$ in direct conflict with Equation (37a), a consequence of the second law. Such a theory then violates the second law as first pointed out by Goldstein [44]; we will revisit this issue in the final section.

We now prove the following theorem:

**Theorem** **6.** For a glass, we must have $H\left(T_{0},P_{0},t\right)>H_{\mathrm{SCL}}\left(T_{0},P_{0}\right)$ at all $T_{0}<$$T_{0g}$, where $S>S_{\mathrm{SCL}}.$

**Proof.**  According to Equations (15) and (37b), we conclude that $d_{e}Q=T_{0}$$d_{e}S<0$ (cf. Equation (46)), while relaxation is going on and vanishes as $T\left(t\right)\to T_{0}^{+}$. It then follows from Equation (43) that:

(56) $$\frac{dH(t)}{dt}\leq 0,$$

a result that is consistent with experimental observations [1]. This completes the proof of the theorem. ☐

It follows from the behavior of the Gibbs free energy $G\left(t\right)=H\left(t\right)-T_{0}S\left(t\right)$ during relaxation (dG(t)/dt≤0) that $dH\leq T_{0}$dS, i.e.,

(57a) $$\left|\frac{dH(t)}{dt}\right|\geq T_{0}\left|\frac{dS(t)}{dt}\right|$$

and:

(57b) $$\left|\Delta H(T)\right|≐H\left(T_{0}\right)-H_{\mathrm{SCL}}\left(T_{0}\right)\geq T_{0}\left[S\left(T_{0}\right)-S_{\mathrm{SCL}}\left(T_{0}\right)\right];T_{0}<T_{0g}.$$

The equality holds at $T_{0}=T_{0g}$. We can also obtain Equation (57a) using $dH=T_{0}$$d_{e}S\leq T_{0}$dS.

From Equations (41) and (37), we also have:

(57c) $$\left|\frac{dH(t)}{dt}\right|\geq T\left(t\right)\left|\frac{dS(t)}{dt}\right|.$$

The last bound is tighter than the bound in Equation (57a) and reduces to the equality obtained earlier [24] where ξ was neglected. This equality there was used to infer Equation (54). We have just established that the conclusion remains unaltered even if we consider internal variables.

In summary, the isothermal relaxation originates from the tendency of the glass to come to thermal equilibrium during which its temperature T(t) approaches $T_{0}$ from above in time. The relaxation process results in the lowering of the corresponding Gibbs free energy in time, as expected due to the second law. However, it also results in the lowering of the corresponding entropy as shown in Figure 3 and the enthalpy during vitrification; the latter is observed experimentally [1].

## 8. Temperature Disparity due to Fast and Slow Variables: Tool–Narayanaswamy Equation

We have shown that for a given $\tau_{\mathrm{obs}}$, we can partition ξ into two distinct groups: one containing internal variable $\xi_{E}$ whose affinity has vanished and the other one, which we now denote by $\xi_{N}$, that has not equilibrated and has a nonzero affinity A. These are the active internal variables. As $\xi_{E}$ has equilibrated, its temperature, pressure, etc., must be those of the medium, that is $T_{0},P_{0}$, etc. It is the inactive internal variable. On the other hand, the temperature, pressure, etc., associated with different components of $\xi_{N}$ must not be those of the medium as there will be nonzero thermodynamic forces to bring each to equilibrium in due course. This raises a very interesting question. Because we are dealing with an IEQ state of the system, there is a well-defined and unique thermodynamic definition of its temperature T(t)≐∂E(t)/∂S(t). This temperature also satisfies the identity dQ(t)=T(t)dS(t). How does T(t) relate to the temperatures of $\xi_{E}$ and $\xi_{N}$? To make some progress, we assume $\xi_{E}$ and $\xi_{N}$ to be quasi-independent over $\tau_{\mathrm{obs}}$. There is strong experimental evidence for this [82,83]. However, there are observables in X that also participate in relaxation. For example, *V* will relax if $P\neq P_{0}$. Similarly, *E* will relax if $T\neq T_{0}$. As we have discussed earlier [24], one can treat E,V, etc., in X as internal variables with their affinities $1/T-1/T_{0},P/T-P_{0}/T_{0}$, etc., that vanish once equilibrium is reached. This is also seen from Equation (14), where the first two terms have the same form as the last term involving dξ; recall that $dS_{0}/dt=d_{i}S/dt$. Therefore, in this section, we will continue to include $\xi_{0}=X$ in ξ as we had done in Section 4. This should not cause any confusion. We only have to be careful to always include $\xi_{0}=X$ to specify the system even when $\tau_{\mathrm{obs}}>$ $\tau_{\mathrm{eq}}=\tau_{0}$.

### 8.1. A Black Box Model

We consider a simple NEQ laboratory problem to model the above situation. Consider a system as a “black box” consisting of two parts at different temperatures $T_{1}$ and $T_{2}>T_{1}$, but insulated from each other so that they cannot come to equilibrium. The two parts are like slow and fast motions in a glass or $\xi_{E}$ and $\xi_{N}$, and the insulation allows us to treat them as independent, having different temperatures. We assume that there are no irreversible processes that go on within each part so that there is no irreversible heat $d_{i}Q_{1}$ and $d_{i}Q_{2}$ generated within each part. We wish to identify the temperature of the system, the black box. To do so, we imagine that to each part is added a certain infinitesimal amount of heat from outside, which we denote by $dQ_{1}=d_{e}Q_{1}$ and $dQ_{2}=d_{e}Q_{2}$. We assume the entropy changes to be $dS_{1}$ and $dS_{2}$. Then, we have for the net heat and entropy change:

$$dQ=dQ_{1}+dQ_{2},dS=dS_{1}+dS_{2}.$$

We introduce the temperature *T* by dQ=TdS. This makes it a thermodynamic temperature of the black box; see Equation (33). Using $dQ_{1}=T_{1}dS_{1},dQ_{2}=T_{2}dS_{2}$, we immediately find:

$$dQ\left(1/T-1/T_{2}\right)=dQ_{1}\left(1/T_{1}-1/T_{2}\right).$$

By introducing $x=dQ_{1}/dQ$, which is determined by the setup, we find that *T* is given by:

(58) $$\frac{1}{T}=\frac{x}{T_{1}}+\frac{1-x}{T_{2}}.$$

As *x* is between zero and one, it is clear that *T* lies between $T_{1}$ and $T_{2}$ depending on the value of *x*. Thus, we see from this heuristic model calculation that the thermodynamic temperature *T* of the system is not the same as the temperature of either parts, a common property of a system not in equilibrium.

If the insulation between the parts is not perfect, there is going to be some energy transfer between the two parts, which would result in maximizing the entropy of the system. As a consequence, their temperatures will eventually become the same. During this period, *T* will also change until all three temperatures become equal.

### 8.2. Tool–Narayanaswamy Equation

We turn to the general case of the relaxation of thermodynamic properties. At high enough temperatures, the time variation of T(t) as it relaxes towards $T_{0}$ can be described as a single simple exponential with a characteristic time scale $\tau_{\mathrm{eq}}$. This happens when all internal variables have come to equilibrium during $\tau_{\mathrm{obs}}>$ $\tau_{\mathrm{eq}}$, so no internal variables besides $\xi_{0}$ are needed, a case discussed by Landau and Lifshitz [7] and by Wilks [10].

At low temperatures, this is not true. There are quasi-independent slow and fast internal variables $\xi_{N}$ and $\xi_{E}$ that are well known in glasses and supercooled liquids [82,83]. The situation is similar to the black box considered above. Both parts will strive to come to equilibrium with the medium, but they have widely separated relaxation times. As time goes on during relaxation, some of the groups in $\left\{\xi_{n}\right\}$ introduced in Section 4 become part of $\xi_{E}$ after equilibration, as we have discussed there. We first assume, for simplicity, that all active internal variables in $\xi_{N}$ have the same relaxation time $\tau_{1}$, i.e., they equilibrate together, but have not equilibrated. The quasi-independence of $\xi_{N}$ and $\xi_{E}$ immediately leads to the following partition of the S,E,V and ξ into two contributions, one from each kind:

(59) $$Z\left(t\right)=Z_{E}\left(t\right)+Z_{N}\left(t\right).$$

For example, quasi-independence gives the additivity $S\left(t\right)=S_{E}\left(t\right)+S_{N}\left(t\right)$, where $S_{E}\left(t\right)$ and $S_{N}\left(t\right)$ stand for $S_{E}\left(E_{E}\left(t\right),V_{E}\left(t\right),\xi_{E}\left(t\right)\right)$ and $S_{N}\left(E_{N}\left(t\right),V_{N}\left(t\right),\xi_{N}\left(t\right)\right)$, etc. Here, we have introduced $V_{E}\left(t\right)$ as the volume difference $V-V_{f}\left(t\right)$ in terms of the free volume $V_{f}\left(t\right)$ in the cell model in which $V_{f}\left(t\right)$ allows for the molecules to move long distances (liquid-like slow motion) over $\tau_{\mathrm{obs}}$ [84]. Thus, $V_{E}$ corresponds to the fast center of mass solid-like motion within the cells, which are in equilibrium with the medium; see also Zallen [72].

Let us now introduce the “energy fraction” x(t) as:

(60) $$x\left(t\right)\equiv dE_{N}\left(t\right)/dE\left(t\right),\;1-x\left(t\right)\equiv dE_{E}\left(t\right)/dE\left(t\right),\;$$

at a given *t*, so that:

(61) $$\partial S_{N}\left(t\right)/\partial E\left(t\right)=x\left(t\right)\partial S_{N}\left(t\right)/\partial E_{N}\left(t\right),\;\partial S_{E}\left(t\right)/\partial E\left(t\right)=\left[1-x\left(t\right)\right]\partial S_{E}\left(t\right)/\partial E_{E}\left(t\right).$$

By definition, we have $\partial S_{E}\left(t\right)/\partial E_{E}\left(t\right)=1/T_{0}$, while $\xi_{N}$ will have a temperature different from this. Assuming internal equilibrium, we can introduce a new temperature $T_{N}\left(t\right)$ by:

(62) $$\partial S_{N}\left(t\right)/\partial E_{N}\left(t\right)=1/T_{N}\left(t\right).$$

The following identity:

(63) $$\frac{1}{T(t)}=\frac{1-x(t)}{T_{0}}+\frac{x(t)}{T_{N}\left(t\right)}$$

easily follows from considering ∂S(t)/∂E(t) and using Equation (59) for S(t) and Equation (61). This equation should be compared with (58) obtained above using a black box model and is identical to the Tool–Narayanaswamy equation [1] in form, except that we have given thermodynamic definitions of x(t) in (60) and $T_{N}\left(t\right)$ in Equation (62).

It is easy to extend the above calculation to the case of different groups $\left\{\xi_{n}\right\}$ belonging to $\xi_{N}$. The quasi-independence gives:

(64) $$Z\left(t\right)=Z_{E}\left(t\right)+\sum_{n}Z_{n}\left(t\right),$$

so that $S\left(t\right)=S_{E}\left(t\right)+\sum_{n}S_{n}\left(t\right)$ with $S_{E}\left(E_{E}\left(t\right),V_{E}\left(t\right),\xi_{E}\left(t\right)\right)$ and $S_{n}\left(E_{n}\left(t\right),V_{n}\left(t\right),\xi_{\neg n}\left(t\right)\right)$ as discussed above. For each $S_{n}$, we have its own temperature $T_{n}$ using. It is now easy to see that Equation (63) is extended to:

(65) $$\frac{1}{T(t)}=\frac{1-x(t)}{T_{0}}+\sum_{n}\frac{x_{n}\left(t\right)}{T_{n}\left(t\right)},$$

with $x_{n}\left(t\right)\equiv dE_{n}\left(t\right)/dE\left(t\right)$ and $1-x\left(t\right)=\sum_{n}x_{n}\left(t\right)$.

Let us now understand the significance of the above analysis. The partition in Equations (59) and (64) along with the fractions x(t) and $x_{n}\left(t\right)$ shows that the partition satisfies a lever rule: the relaxing glass can be conceptually (but not physically) thought of as a “mixture” consisting of different “parts” corresponding to different temperatures and fractions. However, one of the temperatures is $T_{0}$ of the medium, while $T_{n}\left(t\right)$’s denote the temperature of the parts that are not equilibrated yet. As some of these parts equilibrate, their temperature becomes $T_{0}$, and they add to the weight 1−x(t) for the equilibrated internal variables. Thus, we see that while $\xi_{E}\left(t\right)$ may play no role in the IEQ thermodynamics, it still plays an important role in relating the thermodynamic temperature T(t) with those of various groups of ξ(t). Thinking of a system conceptually as a “mixture” of “parts” is quite common in theoretical physics. One common example is that of a superfluid, which can be thought of as a “mixture” of a normal viscous “component” and a superfluid “component”. In reality, there exist two simultaneous motions, one of which is “normal”, and the other one is “superfluid”. A similar division can also be carried out in a superconductor: the total current is a sum of a “normal current” and a “superconducting current”.

Such an analysis has been carried out in detail earlier [24], where a connection is made with the notion of the “fictive” temperature [1], but in the absence of any internal variables (besides $\xi_{0}$). Here, we will summarize that discussion and refer the reader to this work for missing details. It is easy to first consider the simple case in Equation (63). One can consider the part $\xi_{N}$ of the energy fraction x(t) at $T_{N}$ to represent a “fictitious” SCL at temperature $T_{N}$. It is fictitious since the entire system does not consist of this part, so it is not in equilibrium as SCL is supposed to be; it is missing the part corresponding to the fraction 1−x(t). We can supplement mentally the fictitious SCL by the same SCL of fraction x(t) at the same temperature $T_{N}$ to ensure that the entire system consists of $\xi_{N}$ at $T_{N}$. This now represents an IEQ state at $T_{N}$, the left side of Equation (63). Thus, $T_{N}$ represents the thermodynamic temperature of this IEQ state, which can then be treated as an “unequilibrated” SCL, in thermal equilibrium with a medium at $T_{N}$ (but not at $T_{0}$). We have identified it as an “unequilibrated” SCL since there is no reason for $A_{N}$ corresponding to $\xi_{N}$ to vanish in this SCL, whereas it is required to vanish in equilibrium. This SCL at $T_{N}$ is also not identical to the glass as the latter has $\xi_{E}\left(t\right)$ at $T_{0}$, which is absent in this SCL. We can thus justify $T_{N}$ as the fictive temperature.

This picture can be extended to Equation (65) by introducing $T_{N}$ as follows:

$$\frac{x(t)}{T_{N}}=\sum_{n}\frac{x_{n}\left(t\right)}{T_{n}\left(t\right)},$$

which converts it to Equation (63). We can then introduce an equilibrated SCL, in equilibrium with a medium at $T_{N}$ so that we can treat $T_{N}$ as the fictive temperature.

Instead of considering a derivative of *S* with *E*, we can consider derivatives with respect to other state variables such as *V*. In that case, a similar analysis can be carried out as done in [24] to obtain a similar looking Tool–Narayanaswamy equation for P(t)/T(t). We leave it to the reader to carry out this simple extension. The result for $P=P_{0}$ is given in [24].

## 9. Discussions and Conclusions

### 9.1. Consequence of the Relaxation Hierarchy

We have presented a hierarchical classification of relaxation times in increasing order in Equation (17), which allows us to determine a unique temporal window $\Delta t_{n}$ in Equation (19) for a given $\tau_{\mathrm{obs}}$ as shown by the two neighboring relaxation curves around the red horizontal line at the temperature $T_{0}$ of interest in Figure 2. The discussion is valid for any relaxing system with complex relaxation and is not restricted to only SCL/glass undergoing vitrification. The temporal window is not fixed as the state of the system changes, so it must be adjusted appropriately; see Figure 2. Let us consider the vitrification considered in Section 6. Above $T_{0}=T_{0g}$, the system is always in equilibrium (recall that we have used SCL as the equilibrium state) so $\tau_{\mathrm{obs}}\geq \tau_{0}$; see Equation (22). There are no active internal variables. Therefore, the system’s temperature $T=T_{0}$. Slightly below $T_{0g}$, but above $T_{01}$, Equation (21) is satisfied, so $\xi_{0}=X$ is active, but all $\xi_{k},k\geq 1$ are inactive, so they need not be considered for a thermodynamic description. There are two different contributions that affect the temporal window that needs to be considered:

(i) Cooling effect: As we lower $T_{0}$ from its previous value $T_{0}^{\prime }=T_{0g}$ ($\tau_{\mathrm{obs}}=\tau_{0}$), the system’s initial temperature is $T\left(0\right)=T_{0}^{\prime }$. As the system’s temperature determines $\tau_{1}$, it has the previous value $\tau_{1}^{\prime }$ at $T_{0}^{\prime }$ initially, so it lies below the curve $\tau_{1}$ at $T_{0}$. However, the value of $\tau_{0}$ at $T_{0}$ is determined by the new temperature $T_{0}$, so it increases compared to $\tau_{0}^{\prime }=\tau_{0}\left(T_{0}^{\prime }\right)$. Consequently, we have $\tau_{\mathrm{obs}}<\tau_{0}$ to satisfy Equation (21).
(ii) Relaxation effect: During isothermal relaxation at the new temperature, T(t) decreases towards $T_{0}$, which increases $\tau_{1}$ from $\tau_{1}^{\prime }=\tau_{1}\left(T_{0}^{\prime }\right)$ to $\tau_{1}\left(T_{0}\right)$. This shrinks the window $\Delta t_{0}$ in Equation (21) in width to the width shown in Figure 2 at $T_{0}$.

The discussion can be now applied to the sequence of cooling steps to $T_{0}$ between $T_{01}$ and $T_{02}$, between $T_{02}$ and $T_{03}$, etc., where we are confronted with the new successive windows $\Delta t_{1},\Delta t_{2},\ldots$. In each window, we need to consider newer internal variables $\xi_{1},\xi_{2},\ldots$ so that in a window $\Delta t_{n}$, we need to consider $Z_{n}$ consisting of X and $\xi^{\left(n\right)}$ as discussed in Section 4. We thus conclude that the dimension of the state space continues to grow during cooling until all internal variables (presumably leaving out $\xi_{v}$ that refers to local vibrations as noted earlier) become active. Thus, in the glass transition region between $T_{0G}$ and $T_{0g}$, the irreversibility continues to grow until all internal variables become active.

### 9.2. Residual Entropy

As discussed above, we cannot just consider a fixed, small number of internal variables (their number keeps changing in the transition region) if we want to go to some small enough temperatures $T_{0}<T_{0G}$ and be able to describe the cooling process thermodynamically. The best we can do is to determine a large enough number of the internal variables that become active in the transition region. This requires a deeper understanding of the structure of glasses and identifying these internal variables, which seems to be an impossible task at present. In our view, this remains an unsolved problem at present. Despite this, the inequalities in Equations (45), (50) and (53) remain valid for any choice of Z.

As these inequalities are very important, we summarize them for the benefit of the reader. According to Equation (45), the residual entropy $S_{R}$ cannot be less than the experimentally-measured or extrapolated $S_{\mathrm{expt}}\left(0\right)$ at absolute zero; the latter itself cannot be less than the entropy of the supercooled liquid at absolute zero. As we have assumed $S_{\mathrm{SCL}}\left(0\right)=0$, we claim the strict inequality:

$$S_{R}>0.$$

Indeed, the strict inequality between $S_{\mathrm{expt}}\left(0\right)$ and $S_{\mathrm{SCL}}\left(0\right)$ holds at all positive temperatures $T_{0}<T_{0g}$, as derived in Equation (50).

We have not discussed the statistical formulation of the residual entropy, which has been discussed by us in [32] (see Section 4.3.3) and [53] (see Section 7). The derivation does not require the use of the second law or entropy maximization. Therefore, it applies to any nonequilibrium state and is purely combinatorial in nature. For the sake of completeness, we summarize the result. Let $\Gamma_{\lambda },\lambda =1,2,\ldots ,C$ denote the number of disjoint components in the state space, and let $p_{k_{\lambda }}$ denote the probability of a microstate $k_{\lambda }$ in $\Gamma_{\lambda }$. The entropy $S=-\sum_{\lambda }\sum_{k_{\lambda }}p_{k_{\lambda }}\ln p_{k_{\lambda }},$$\sum_{\lambda }\sum_{k_{\lambda }}p_{k_{\lambda }}=1$, can be written as a sum of two parts:

$$S=\sum_{\lambda }p_{\lambda }S_{\lambda }+S_{C},$$

where $p_{\lambda }≐\sum_{k_{\lambda }}p_{k_{\lambda }}$ is the probability of the component $\Gamma_{\lambda }$, $S_{\lambda }=-\sum_{k_{\lambda }}{\hat{p}}_{k_{\lambda }}\ln{\hat{p}}_{k_{\lambda }},{\hat{p}}_{k_{\lambda }}≐p_{k_{\lambda }}/p_{\lambda }$, is the entropy of the component, and:

(66) $$S_{C}≐-\sum_{\lambda }p_{\lambda }\ln p_{\lambda }$$

is the component confinement entropy. The residual entropy is the component confinement entropy $S_{C}$ at absolute zero, with $p_{\lambda }=p_{\lambda 0}$ denoting the probability of the component $\Gamma_{\lambda }$ at absolute zero. We have not imposed any equally-probable assumption in the above derivation so the result is very general. However, to apply IEQ thermodynamics, we need to impose an equally-probable assumption.

### 9.3. Fate of the Entropy Loss Conjecture

The isothermal relaxation considered in Section 7 shows that both *S* and *H* decrease with time, which is consistent with our intuitive picture given at the start of that section for *S* and experimental evidence for *H*. As we have shown, the behavior is a consequence of the second law. The entropy loss view (ELV) mentioned after Theorem 5 and proposed in [78,79,80,81] results in a conclusion that contradicts our results. In particular, the view suggests that during relaxation, the entropy increase since $S\left(T_{0},t\right)\leq S_{\mathrm{SCL}}\left(T_{0}\right)$; see Equation (55). As Goldstein [44] has shown, this is a violation of the second law. These authors agree that in their view of the glass transition, the glasses do violate the second law, while others [30,35,74,75,76,77] argue in favor of the second law. For most scientists, the fact that the entropy loss view violates the second law should be a strong indication that the view is unrealistic. However, the debate persists as is evident from some recent reviews [85,86,87,88,89].

Here, we hope to settle the debate by pointing out a hitherto unrecognized internal inconsistency of the ELV, assuming its premise that the glasses do violate the second law. In other words, the second law is not the absolute truth of Nature. This means that all the inequalities in Equation (37) must be reversed for the view to hold. Since dS>0 in ELV during relaxation, it follows from the reverse inequality in Equation (37a) that $T\left(t\right)>T_{0}$, which is the same as Equation (15). From the reverse inequality in Equation (37b), we conclude that:

(67) $$[T_{0}-T\left(t\right)]dH\left(t\right)<0.$$

If we demand that the ELV follow the experimental evidence (dH(t)/dt<0), we must conclude that $T_{0}>T\left(t\right)$, which contradicts the previous conclusion, and the ELV becomes internally inconsistent. If, however, we accept the previous conclusion $T\left(t\right)>T_{0}$ to ensure that the ELV remain internally consistent, then dH(t)/dt>0, in contradiction with experimental evidence. Thus, the mere fact that the ELV satisfies the experimental evidence (dH(t)/dt<0) does not mean that it is internally consistent in the entropy loss view. In other words, demanding that the ELV is consistent with experiments disproves the ELV conjecture. Even though we have considered the entropy loss view at different times [30,53,64,65,66,67,68], we believe that the conclusion drawn above is the most direct demonstration of the internal inconsistency of the ELV, despite the fact that we have allowed it to contradict the second law.

### 9.4.Significance of Inactive Internal Variables

Even though the IEQ thermodynamics only involves the active internal variables, it is clear from Section 8 that even inactive internal variables such as $\tau_{r}$ indirectly affect the thermodynamics through the determination of the temperature, pressure, etc., of the system. In retrospect, this is not so surprising once we recognize that the temperature of the system is a thermodynamic quantity. However, the division of the internal variables into active and inactive parts means that the temperature of the system must be different from the temperature $T_{0}$ during isothermal relaxation.
